# Supplementary material for: Seroprevalence of Chikungunya virus and living conditions in Feira de Santana, Bahia-Brazil
Source: PLoS Negl Trop Dis. 2021 Apr 20;15(4):e0009289. doi: 10.1371/journal.pntd.0009289 (PMC8087031; doi:10.1371/journal.pntd.0009289)
Supplement: S2 Strobe Checklist — (DOC) [file pntd.0009289.s002.doc]

STROBE Statement—Checklist of items that should be included in reports of ***cross-sectional studies***

|  | Item No | Recommendation |
| --- | --- | --- |
| **Title and abstract** | 1 | (*a*) Indicate the study’s design with a commonly used term in the title or the abstract  Seroprevalence of Chikungunya virus and living conditions in Feira de Santana, Bahia-Brazil |
|  |  | (*b*) Provide in the abstract an informative and balanced summary of what was done and what was found  Abstract section |
| Introduction | | |
| Background/rationale | 2 | Explain the scientific background and rationale for the investigation being reported: Introduction, third paragraph |
| Objectives | 3 | State specific objectives, including any prespecified hypotheses: Introduction, third paragraph |
| Methods | | |
| Study design | 4 | Present key elements of study design early in the paper: Design and local of study section, first paragraph |
| Setting | 5 | Describe the setting, locations, and relevant dates, including periods of recruitment, exposure, follow-up, and data collection: Design and local of study section, first paragraph; data collect section, first paragraph and blood collect section, first paragraph |
| Participants | 6 | (*a*) Give the eligibility criteria, and the sources and methods of selection of participants: Data collect section, first paragraph |
| Variables | 7 | Clearly define all outcomes, exposures, predictors, potential confounders, and effect modifiers. Give diagnostic criteria, if applicable: Blood collect and laboratory procedures section, second paragraph |
| Data sources/ measurement | 8* | For each variable of interest, give sources of data and details of methods of assessment (measurement). Describe comparability of assessment methods if there is more than one group Blood collect and laboratory procedures section, second paragraph |
| Bias | 9 | Describe any efforts to address potential sources of bias: Data collect section, first paragraph |
| Study size | 10 | Explain how the study size was arrived at: Determination of sample size section, first and second paragraph |
| Quantitative variables | 11 | Explain how quantitative variables were handled in the analyses. If applicable, describe which groupings were chosen and why: Areas selection section, first, second and third paragraphs |
| Statistical methods | 12 | (*a*) Describe all statistical methods, including those used to control for confounding: Data analysis section, first paragraph |
|  |  | (*b*) Describe any methods used to examine subgroups and interactions: Data analysis section, first paragraph |
|  |  | (*c*) Explain how missing data were addressed: Not applied |
|  |  | (*d*) If applicable, describe analytical methods taking account of sampling strategy Not applied |
|  |  | (*e*) Describe any sensitivity analyses Not applied |
| Results | | |
| Participants | 13* | (a) Report numbers of individuals at each stage of study—eg numbers potentially eligible, examined for eligibility, confirmed eligible, included in the study, completing follow-up, and analysed: Results, first paragraph, Figure 2 |
|  |  | (b) Give reasons for non-participation at each stage: Results, first paragraph; Figure 2 |
|  |  | (c) Consider use of a flow diagram: Figure 2 |
| Descriptive data | 14* | (a) Give characteristics of study participants (eg demographic, clinical, social) and information on exposures and potential confounders: Results, second paragraph; Table 1 |
|  |  | (b) Indicate number of participants with missing data for each variable of interest: Results, second paragraph; Table 1 |
| Outcome data | 15* | Report numbers of outcome events or summary measures: Results, third paragraph; Table 2 |
| Main results | 16 | (*a*) Give unadjusted estimates and, if applicable, confounder-adjusted estimates and their precision (eg, 95% confidence interval). Make clear which confounders were adjusted for and why they were included. Results, third paragraph; Table 2 |
|  |  | (*b*) Report category boundaries when continuous variables were categorized. Results, fourth paragraph |
|  |  | (*c*) If relevant, consider translating estimates of relative risk into absolute risk for a meaningful time period: Not applied |
| Other analyses | 17 | Report other analyses done—eg analyses of subgroups and interactions, and sensitivity analyses: Results, fifth paragraph. |
| Discussion | | |
| Key results | 18 | Summarise key results with reference to study objectives: Discussion, first paragraph |
| Limitations | 19 | Discuss limitations of the study, taking into account sources of potential bias or imprecision. Discuss both direction and magnitude of any potential bias Discussion, nineth paragraph |
| Interpretation | 20 | Give a cautious overall interpretation of results considering objectives, limitations, multiplicity of analyses, results from similar studies, and other relevant evidence: Discussion, second to tenth paragraph |
| Generalisability | 21 | Discuss the generalisability (external validity) of the study results: Discussion, nineth paragraph |
| Other information | | |
| Funding | 22 | Give the source of funding and the role of the funders for the present study and, if applicable, for the original study on which the present article is based: Section “Financial supporters” |

*Give information separately for exposed and unexposed groups.

**Note:** An Explanation and Elaboration article discusses each checklist item and gives methodological background and published examples of transparent reporting. The STROBE checklist is best used in conjunction with this article (freely available on the Web sites of PLoS Medicine at http://www.plosmedicine.org/, Annals of Internal Medicine at http://www.annals.org/, and Epidemiology at http://www.epidem.com/). Information on the STROBE Initiative is available at www.strobe-statement.org.
