# Supplementary material for: Seroprevalence of Chikungunya virus and living conditions in Feira de Santana, Bahia-Brazil
Source: PLoS Negl Trop Dis. 2021 Apr 20;15(4):e0009289. doi: 10.1371/journal.pntd.0009289 (PMC8087031; doi:10.1371/journal.pntd.0009289)
Supplement: Attachment — Submitted filename: PNTD-D-20-01278_reviewer_IW2.pdf [file pntd.0009289.s004.pdf]

PLOS Neglected Tropical Diseases
Seroprevalence of Chikungunya virus and living conditions in Feira de Santana, Bahia-Brazil
--Manuscript Draft--

**Manuscript Number:**	PNTD-D-19-02186R1
**Full Title:**	Seroprevalence of Chikungunya virus and living conditions in Feira de Santana, Bahia-Brazil
**Short Title:**	CHIKV Seroprevalence and living conditions, Brazil
**Article Type:**	Research Article
**Keywords:**	Chikungunya, Seroprevalence, Living conditions, Epidemiology.
**Abstract:**	Background<br>Chikungunya is an arbovirus, transmitted by Aedes mosquitoes, which emerged in the Americas in 2013 and spread rapidly to almost every country on this continent. In Brazil, where the first cases were detected in 2014, it currently have reached all regions of this country and more than 900,000 cases were reported. The clinical spectrum of chikungunya ranges from an acute self-limiting form to disabling chronic forms. The purpose of this study was to estimate the seroprevalence of chikungunya infection in a large Brazilian city and investigate the association between viral circulation and living condition.<br><br>Methodology/Principal findings<br>We conducted a population-based ecological study in selected Sentinel Areas (SA) through household interviews and a serologic survey in 2016/2017. The sample was of 1,981 individuals randomly selected. The CHIKV seroprevalence was 22.1% (17.1 IgG, 2.3 IgM, and 1.4 IgG and IgM) and varied between SA from 2.0% to 70.5%. The seroprevalence was significantly lower in SA with high living conditions compared to SA with low living condition. There was a positive association between CHIKV seroprevalence and population density (r=0.2389; p=0.02033).<br><br>Conclusions/Significance<br>The seroprevalence in this city was 2.6 times lower than the 57% observed in a study conducted in the epicentre of the CHIKV epidemic of this same urban centre. So, the herd immunity in this general population, after four years of circulation of this agent is relatively low. It indicates that CHIKV transmission may persist in that city, either in endemic form or in the form of a new epidemic, because the vector infestation is persistent. Besides, the significantly lower seroprevalences in SA of higher Living Condition suggest that beyond the surveillance of the disease, vector control and specific actions of basic sanitation, the reduction of the incidence of this infection also depends on the improvement of the general living conditions of the population.
**Additional Information:**	
**Question**	**Response**
**Financial Disclosure**<br><br>Enter a financial disclosure statement that describes the sources of funding for the work included in this submission. Review the submission guidelines for detailed requirements. View published research articles from *PLOS NTDs* for specific examples.<br><br><br>This statement is required for submission and **will appear in the published article** if	The funders had no role in study design, data collection and analysis, decision to publish, or preparation of the manuscript.

Powered by Editorial Manager® and ProduXion Manager® from Aries Systems Corporation

the submission is accepted. Please make sure it is accurate.<br><br>**Unfunded studies**<br><br>Enter: *The author(s) received no specific funding for this work.*<br><br>**Funded studies**<br><br>Enter a statement with the following details:<br>• Initials of the authors who received each award<br>• Grant numbers awarded to each author<br>• The full name of each funder<br>• URL of each funder website<br>• Did the sponsors or funders play any role in the study design, data collection and analysis, decision to publish, or preparation of the manuscript?<br>• **NO** - Include this sentence at the end of your statement: *The funders had no role in study design, data collection and analysis, decision to publish, or preparation of the manuscript.*<br>• **YES** - Specify the role(s) played.<br><br>* typeset	
**Competing Interests**<br><br>Use the instructions below to enter a competing interest statement for this submission. On behalf of all authors, disclose any competing interests that could be perceived to bias this work—acknowledging all financial support and any other relevant financial or non-financial competing interests.<br><br>This statement **will appear in the published article** if the submission is accepted. Please make sure it is accurate. View published research articles	The authors have declared that no competing interests exist.

from *PLOS NTDs* for specific examples.<br><br>**NO authors have competing interests**<br><br>Enter: *The authors have declared that no competing interests exist.*<br><br>**Authors with competing interests**<br><br>Enter competing interest details beginning with this statement:<br><br>*I have read the journal's policy and the authors of this manuscript have the following competing interests: [insert competing interests here]*<br><br>* typeset	
**Data Availability**<br><br>Authors are required to make all data underlying the findings described fully available, without restriction, and from the time of publication. PLOS allows rare exceptions to address legal and ethical concerns. See the PLOS Data Policy and FAQ for detailed information.<br><br>A Data Availability Statement describing where the data can be found is required at submission. Your answers to this question constitute the Data Availability Statement and **will be published in the article**, if accepted.	Yes - all data are fully available without restriction

**Important:** Stating 'data available on request from the author' is not sufficient. If your data are only available upon request, select 'No' for the first question and explain your exceptional situation in the text box.<br><br>Do the authors confirm that all data underlying the findings described in their manuscript are fully available without restriction?	
**Describe where the data may be found in full sentences. If you are copying our sample text, replace any instances of XXX with the appropriate details.**<br><br>• If the data are **held or will be held in a public repository**, include URLs, accession numbers or DOIs. If this information will only be available after acceptance, indicate this by ticking the box below. For example: *All XXX files are available from the XXX database (accession number(s) XXX, XXX.*).<br>• If the data are all contained **within the manuscript and/or Supporting Information files**, enter the following: *All relevant data are within the manuscript and its Supporting Information files.*<br>• If neither of these applies but you are able to provide **details of access elsewhere**, with or without limitations, please do so. For example:<br><br>*Data cannot be shared publicly because of [XXX]. Data are available from the XXX Institutional Data Access / Ethics Committee (contact via XXX) for researchers who meet the criteria for access to confidential data.*<br><br>*The data underlying the results presented in the study are available*	All relevant data are within the manuscript and its Supporting Information files

from (include the name of the third party and contact information or URL).<br>• This text is appropriate if the data are owned by a third party and authors do not have permission to share the data.<br><br>* typeset	
Additional data availability information:	

Powered by Editorial Manager® and ProduXion Manager® from Aries Systems Corporation

Manuscript Click here to access/download;Manuscript;plos0308_clean.doc

Seroprevalence of Chikungunya virus and living conditions in Feira de Santana, Bahia-Brazil

Running Head: CHIKV Seroprevalence and living conditions, Brazil

Maria Glória Teixeira [1]
Lacita Menezes Skalinski [1,2]
Enny S Paixão[3]
Maria da Conceição N Costa[1]
Florisneide Rodrigues Barreto[1]
Gubio Soares Campos[4]
Silvia Ines Sardi[4]
Rejane Hughes Carvalho[4]
Marcio Natividade[1]
Martha Itaparica[1]
Juarez Pereira Dias[1]
Soraya Castro Trindade[5]
Bárbara Pereira Teixeira[1]
Vanessa Morato[6]
Eloisa Bahia Santana[7]
Cristina Borges Goes[8]
Neuza Santos Jesus[7]
Carlos Antonio de Souza Teles Santos[9]
Laura C Rodrigues[3]
Jimmy Whitworth [3]

Author for correspondence:

Maria Gloria Teixeira, Rua Basílio da Gama, s/n. Campus Canela, Salvador/BA/BR CEP 40110-040.
magloria@ufba.br, Tel +557132837445; Fax: +557132837445

1. Instituto de Saúde Coletiva/ Universidade Federal da Bahia
2. Departamento de Ciências da Saúde / Universidade Estadual de Santa Cruz
3. London School of Hygiene and Tropical Medicine
4. Instituto de Ciências da Saúde / Universidade Federal da Bahia
5. Programa de Pós-graduação em Biotecnologia / Universidade Estadual de Feira de Santana
6. Secretaria de Segurança Pública do Estado da Bahia
7. Secretaria Municipal de Saúde de Feira de Santana
8. Faculdade de Tecnologia e Ciências de Feira de Santana
9. Centro de Pesquisas Gonçalo Muniz/Fiocruz, Salvador-BA

Abstract

Background

Chikungunya is an arbovirus, transmitted by *Aedes* mosquitoes, which emerged in the Americas in 2013 and spread rapidly to almost every country on this continent. In Brazil, where the first cases were detected in 2014, it currently have reached all regions of this country and more than 900,000 cases were reported. The clinical spectrum of chikungunya ranges from an acute self-limiting form to disabling chronic forms. The purpose of this study was to estimate the seroprevalence of chikungunya infection in a large Brazilian city and investigate the association between viral circulation and living condition.

Methodology/Principal findings

We conducted a population-based ecological study in selected Sentinel Areas (SA) through household interviews and a serologic survey in 2016/2017. The sample was of 1,981 individuals randomly selected. The CHIKV seroprevalence was 22.1% (17.1 IgG, 2.3 IgM, and 1.4 IgG and IgM) and varied between SA from 2.0% to 70.5%. The seroprevalence was significantly lower in SA with high living conditions compared to SA with low living condition. There was a positive association between CHIKV seroprevalence and population density (r=0.2389; p=0.02033).

Conclusions/Significance

The seroprevalence in this city was 2.6 times lower than the 57% observed in a study conducted in the epicentre of the CHIKV epidemic of this same urban centre. So, the herd immunity in this general population, after four years of circulation of this agent is relatively low. It indicates that CHIKV transmission may persist in that city, either in endemic form or in the form of a new epidemic, because the vector infestation is persistent. Besides, the significantly lower seroprevalences in SA of higher Living Condition suggest that beyond the surveillance of the disease, vector control and specific actions of basic sanitation, the reduction of the incidence of this infection also depends on the improvement of the general living conditions of the population.

Keywords: Chikungunya, Seroprevalence, Living conditions, Epidemiology.

Author Summary

Chikungunya is a disease caused by viruses transmitted by *Aedes* mosquitoes. It stands out because it can produce chronic cases and disabling forms. This survey was conducted in a large city in Brazil, involving 1,981 people living in different living conditions/LC, distributed in 30 urban areas. Interviews and blood collection were performed to know the seroprevalence of chikungunya virus (CHIKV). The global seroprevalence was 22.1%, ranged from 2.0 to 70.5%. The areas where the highest and lowest seroprevalence were of low LC, but population density was different among them, which was related to seroprevalence. The mosquito index was not associated with the seroprevalence. Our results showed that lower seroprevalence than another survey conducted in the epicentre of the epidemic of this same city. So, a considerable proportion of the population of Feira de Santana is susceptible to the CHIKV. Possibly the virus will continue to circulate and manifest itself as endemic or epidemic, as long as LC remain favourable to the reproduction of the transmitting vector of CHIKV.

Introduction

Chikungunya has stood out among emerging arboviruses, because of its potential to produce epidemics with high rates of chronic and disabling sequeleae, especially arthritis, which can last from months to years.[1] The agent of this disease, chikungunya virus (CHIKV) belonging to *Togaviridae* family, is transmitted by mosquitoes of the genus *Aedes.*[2] Before 2004, human CHIKV infections were restricted to parts of Africa, and in 2005, a major outbreak occurred in islands of the Indian Ocean.[3] Since then, epidemics of chikungunya have been reported in Italy[4], India, Indonesia, Maldives, Myanmar and Thailand[5], and in 2013, chikungunya reached the Americas, where the first autochthonous cases were confirmed in the Caribbean island of St Martin[6], and quickly spread to over 43 territories, causing more than 2.6 million cases by 2017.[7]

There are already many studies about chikungunya seroprevalence, which have results ranging from 0.4% to over 75%. This variation is a reflection of sample selection (random or convenience), the magnitude of epidemics at each site, time of virus circulation before the survey, demographic and environmental characteristics of each site, among other aspects. [8]

Previous studies on dengue, another arbovirus circulating in Brazil, have shown seroprevalence varying by place of residence, reflecting living conditions, such as overcrowding, low education, low income, poor sanitation and high exposure to the infected vectors.[9,10] Although CHIKV is transmitted by the same mosquito as dengue; there is a lack of information regarding the relationship between the occurrence of CHIKV infection and socio-economic conditions in the urban environment. Therefore, this study aims to estimate the seroprevalence of chikungunya infection in a large Brazilian city and investigate the association between viral circulation and living condition, using an ecological approach.

Methods

Design and Local of Study

We conducted a population-based ecological study through household interviews and serological survey from November 2016 to September 2017, involving residents of 30 delimited areas named "Sentinel Areas" (SA) in Feira de Santana (FSA) city, Northeast Brazil (Figure 1). This city is the second most populous in Bahia state,

with 609,913 inhabitants, a population density of 416.03 inhabitants/ $km^{2,}$ and it is 110 km far from Salvador, the capital of Bahia. Located in the transition zone between Zona da Mata (forest zone) and Sertão (dry forest), it has tropical weather with high temperatures and relative humidity ranging from 74 to 88%.[11] Chikungunya virus (CHIKV) serotype ECSA emerged in Brazil in 2014, in FSA[12] where it started to circulate intensely. In FSA, dengue epidemics has occurred for more than two decades and the ZIKV was identified in 2015.[13]

Figure 1. Maps show locations of Feira de Santana in Bahia state and Bahia state in Brazil.

Areas Selection

To select the 30 SA we used the Living Conditions Index (LCI) as a proxy of living conditions (LC), similar to proposed by Paim et al (2003).[14] For this, from the Demographic Census data of the year 2000, these authors used five indicators to the variables income (proportion of householders in permanent private households with average monthly income equal to or less than two minimum wages), slum (percentage of houses in subnormal agglomerate to the total of households in the area), crowding (ratio between the average number of residents per household in the area and the average number of rooms serving dormitory in its area), sanitation (percentage of households with indoor plumbing connected to the global network of water supply) and education (proportion of persons 10 to 14 years old literate) for each Information Zone (IZ), unit of analysis employed in that study. Next, income, slum and subnormal agglomerate indicators were arranged in ascending order while education and sanitation were arranged in descending order considering the average value of each of them in the respective IZ. Then, each indicator received a numerical value started by 1, according to the position assumed in this order and, after that, the values obtained by each one IZ were summed up thus obtaining a score representative of the living conditions of the population of IZ. Subsequently, these scores were sorted in ascending order. Thus, higher scores corresponded to lower living conditions. Next, they were grouped in quartiles ZI corresponding to population segments classified in the following categories of living conditions: high, intermediate, low and very low.

Although for the present study all of these procedures have been adopted, as we used data from 2010 Census that did not have all information required for construction of the slum and crowding indicators, we had to exclude the first one and to build the last one as the “ratio inhabitants per room”. The 30 SA were composed by a grouping of CT and they were selected considering socioeconomic and demographic criteria. Thus, the SA had greater uniformity within those in the same stratum of LC and more considerable heterogeneity between the strata.

The Municipal Health Secretariat of Feira de Santana has an extemsive network of public health services distributed throughout the city's urban territory. As this survey is part of a platform of longitudinal clinical and epidemiological studies on dengue, Zika and chikungunya, we considered important that the population residing in the intra-urban spaces selected for these studies resided in the territories covered by Public Health Units. This way, in the course of the study, the research team would have access to information about the occurrence of cases of these arboviruses. Then, all SA should be close to one Basic Health Unit (BHU) or Family Health Unit (FHU) that offered good quality care for the population. Professionals of Epidemiologic Surveillance of Feira de Santana/FSA city indicated the Basic Health Units (BHU) and the Family Health Units (FHU), which provided better quality care. They selected 46 of the 92 units located in urban FSA, which 14 were BHU. All 14 UBS were considered for the study because they performed complex care, and have health agents on their team. The other 16 SA were selected in CT where the FHU were not located in violent areas and offered good quality care. Health professionals who coordinated these units made this selection. As CT varied greatly in their population size, the SA had minimum 773 and maximum 2,442 people, according to IBGE data. Then, each SA had one, two or three CT with an average of 1,310 people residing in each SA, making a total of 40 CT.

Data Collect

A SA census was conducted by fieldworkers who visited all households in the selected SA and invited all household members from one year old to take part in the study. We excluded those who lived in that household for less than six months. If all residents or any of them were not found at the time of the visit, a second attempt was made at another time or on weekends. Trained interviewers explained the project objectives, and, when allowed, collected participant's signatures in the Free and

Informed Consent Form (FICF) and conducted the interviews. For participants <18 years old, we used the Term of Assent and a responsible adult signed the document.

We used a semi-structured questionnaire, installed on tablets, divided in two sections: a) household section: address of the domicile; names of all residents and household position and household income (In 2010, the national minimum annual wage was approximately US$ 300.00); b) individual section: identification data; socio-demographics; self-reported morbidity, specifically related to the three arboviruses that circulate in FSA (Zika, dengue and chikungunya); presence of co-morbidities and continuous use of medication.

Determination of sample size

The sample design was in a two-stage conglomerate, with the sample fraction of the 1st stage, the sentinel units (f1=1.0) that were self-represented, that is, they were all part of the study, and the sample fraction of the 2nd stage, selection of individuals (f2=f/f1) of 0.05, to compose the sample of each sentinel area, in order to maintain the simple random sampling fraction of 0.05 (f=f1*f2).

To calculate the sample size, we used the simple random sampling formula, with sample fraction f=n/N, considering the prevalence of the specific event of 50%; significance level of 5% and sampling error of 0.025. Thus, the sample size of 1,504 was obtained. This sample was expanded to 2,114 by assuming an effect around 1.42. In addition, we added 40% more in the final sample in order to avoid loss of power due to refusals, resulting in 2,960 individuals who were randomly recruited.

Blood collect and Laboratory Procedures

The blood collection started in April 2017 and concluded in September 2017. After signing the specific FICF, trained laboratory technicians collected 5 mL of blood per venipuncture, in accordance with current biosafety standards. Local laboratory staff separated the serum by centrifugation and stored it at –20°C. We transported the frozen aliquots to the virology laboratory of the Federal University of Bahia/Institute of Health Sciences, where the laboratory tests were processed.

The presence of IgM or IgG antibodies against CHIKV was detected in serum samples using the Chikungunya anti-virus ELISA (IgM and IgG) from Euroimmun (Lübeck, Schleswig-Holstein, Germany), following the manufacturer's instructions[15]. This test is based on a calculation given by the ratio of the coefficient of extinction of the sample and the coefficient of extinction of the calibrator provided in the kit.

Samples with ratio below 0.8 are considered negative, while those above or equal to 1.1 are positive. Samples above or equal to 0.8 and below 1.1 are considered as inconclusive.

Entomological Indicator

The entomological indicator used was the Premise Index (PI)[16] obtained for each SA as the percentage of all inspected premises which were found with at least one positive breeding site for *Aedes aegypti* larvae. These data were provided by professionals of Entomological Surveillance System of the municipality.

Data analysis

We estimated the crude seroprevalence by SA. The global seroprevalence, by LC strata and by sex were estimated and presented the respective Confidence Intervals of 95% and design effect. The ratio seroprevalence by LC was estimated as well as the respective Confidence Intervals of 95%. Pearson chi squared (χ^2) tests were used to test differences between CHIKV seroprevalence and LC strata, assuming $p<0.05$. To test the correlation between CHIKV seroprevalence and LC, population density and PI, we applied the non-parametric Spearman correlation test. All analyses were performed using Stata software version 12.0 (Stata Corporation, College Station, USA).

Ethical aspects

This research was approved by the Committee on Ethics in Research of Institute of Collective Health/Federal University of Bahia, under number 1.685.039/2016.

Results

We visited 15,045 households and 6,977 of these were excluded (being commercial, uninhabited, or refusals). In the remaining 8,068 (53.6%) households, we interviewed 16,620 inhabitants. Next, 2,960 (17.8%) of them were randomly selected to the serosurvey, but only 1,981 (66.9%) agreed to participate (Figure 2), being 67.6% women and almost 25% aged over 60 years. This distribution was similar to that of the interviewed population.

Figure 2. Flowchart of seroprevalence survey of CHIKV in dwellers from 30 Sentinel Areas of Feira de Santana/FSA. Bahia-Brazil, 2017.

The majority of participants self-declared as mixed race (62.2%) or black (23.0%). However, in the SA with high LC there was a larger proportion of white people (19.2%) than in the low LC (8.8%). The percentage of inhabitants reporting more than 12 years of formal education was highest in the high LC area (11.7%) and decreased as the LC declined to the very low area (3.3%). It was observed that in all SA predominated income between 1 and 2 minimum wages and only in the high and intermediate LC strata there were SA with income greater than five minimum wages (Table 1). These differences were statistically significant. The percentage of data missing related to income variable was high in all AS (average 35.1%), ranging from 29.3% to 38.0% in low and high LC, respectively.

Table 1. Sociodemographic characteristics of the population of serological survey for chikungunya by Sentinel Area (SA) and Living Conditions (LC) strata. Feira de Santana – Bahia, April to September, 2017.

LC strata	SA number	Serosurvey population (n)	Race (%)				Education in years of study (%)				Household income in NMW[1] (%)				
			White	Black	Mixed	Others/ missing	<8	8-11	>12	Missing	<1	1-2	2-5	>5	Missing
High	6	50	18.0	18.0	58.0	6.0	34.0	50.0	12.0	4.0	26.0	38.0	8.0	-	28.0
	26	62	19.4	25.8	54.8	-	38.7	48.4	4.8	8.1	14.5	41.9	1.6	-	41.9
	23	60	31.7	3.3	65.0	-	41.7	48.3	10.0	-	8.3	50.0	6.7	-	35.0
	3	68	16.2	16.2	66.2	1.5	36.8	44.1	16.2	2.9	16.2	39.7	13.2	2.9	27.9
	5	41	12.2	4.9	82.9	-	48.8	43.9	7.3	-	22.0	14.6	9.8	2.4	51.2
	8	71	9.9	22.5	66.2	1.4	29.6	47.9	19.7	2.8	14.1	38.0	2.8	1.4	43.7
	**Mean**	**59**	**19.5**	**15.1**	**65.5**	**1.5**	**38.3**	**47.1**	**11.7**	**3.0**	**16.9**	**37.0**	**7.0**	**1.1**	**38.0**
Intermediate	16	47	12.8	38.3	48.9	-	40.4	46.8	10.6	2.1	46.8	17.0	2.1	-	34.0
	27	56	19.6	33.9	42.9	3.6	44.6	48.2	7.1	-	39.3	51.8	3.6	-	5.4
	9	55	9.1	29.1	54.5	7.3	32.7	45.5	20.0	1.8	7.3	61.8	12.7	-	18.2
	1	118	8.5	15.3	75.4	0.8	44.1	49.2	5.1	1.7	19.5	32.2	2.5	-	45.8
	14	82	20.7	18.3	59.8	1.2	37.8	50.0	11.0	1.2	12.2	52.4	7.3	-	28.0
	4	109	16.5	13.8	68.8	0.9	37.6	52.3	4.6	5.5	25.7	43.1	2.8	-	28.4
	24	65	13.8	15.4	70.8	-	50.8	27.7	13.8	7.7	16.9	40.0	6.2	-	36.9
	10	74	12.2	40.5	44.6	2.7	40.5	45.9	6.8	6.8	33.8	18.9	2.7	1.4	43.2
	7	53	9.4	18.9	71.7	-	43.4	45.3	3.8	7.5	47.2	18.9	-	1.9	32.1
	19	56	10.7	21.4	64.3	3.6	37.5	41.1	17.9	3.6	19.6	30.4	5.4	1.8	42.9
	2	71	11.3	25.4	62.0	1.4	38.0	46.5	12.7	2.8	18.3	40.8	5.6	1.4	33.8
	**Mean**	**71**	**13.1**	**24.6**	**60.3**	**2.0**	**40.7**	**45.3**	**10.3**	**3.7**	**26.1**	**37.0**	**4.6**	**0.6**	**31.7**
Low	20	67	14.9	31.3	49.3	4.5	52.2	37.3	7.5	3.0	22.4	34.3	3.0	-	40.3
	28	82	18.3	24.4	54.9	2.4	46.3	42.7	8.5	2.4	58.5	31.7	1.2	-	8.5
	15	61	6.6	37.7	52.5	3.3	70.5	24.6	3.3	1.6	67.2	18.0	-	-	14.8
	29	51	25.5	17.6	52.9	3.9	41.2	41.2	17.6	-	15.7	54.9	9.8	-	19.6
	13	84	9.5	10.7	79.8	-	40.5	44.0	11.9	3.6	23.8	26.2	1.2	-	48.8
	22	70	7.1	34.3	57.1	1.4	45.7	32.9	17.1	4.3	35.7	27.1	1.4	-	35.7
	12	61	9.8	45.9	42.6	1.6	47.5	49.2	-	3.3	41.0	29.5	-	-	29.5
	21	96	8.3	29.2	59.4	3.1	43.8	44.8	1.0	10.4	28.1	32.3	2.1	-	37.5
	**Mean**	**52**	**12.5**	**28.9**	**56.1**	**2.5**	**48.5**	**39.6**	**8.4**	**3.6**	**36.6**	**31.8**	**2.3**	**-**	**29.3**
Very low	17	56	5.4	19.6	73.2	1.8	71.4	21.4	1.8	5.4	32.1	26.8	1.8	-	39.3
	30	70	4.3	28.6	60.0	7.1	60.0	24.3	7.1	8.6	48.6	18.6	-	-	32.9
	25	36	16.7	11.1	69.4	2.8	38.9	52.8	5.6	2.8	8.3	44.4	2.8	-	44.4
	18	52	13.5	11.5	75.0	-	63.5	34.6	1.9	-	44.2	42.3	-	-	13.5
	11	50	4.0	24.0	68.0	4.0	40.0	58.0	-	2.0	8.0	48.0	2.0	-	42.0
	**Mean**	**53**	**8.8**	**19.0**	**69.1**	**3.1**	**54.8**	**38.2**	**3.3**	**3.8**	**28.2**	**36.0**	**1.3**	**-**	**34.4**

(1) National Minimum Wage. One national minimum wage is approximately US$ 300.00.

The global CHIKV seroprevalence was 22.1% (CI95% 16.7-28.6), being 82.3% of these were IgG only; 11.2% IgM only; and 6.5% both IgG and IgM and varied from 2.0% to 70.5% in the SA 29 and 12, respectively, both located in the low stratum (Figure 3A and 3B). The median value of seroprevalence in SA of high LC was 9.9% (3.2 to16.7%), 18.9% (5.4 to 47.7%) in intermediate, 14.1% (2.0 to70,5%) in low and 12.9% (8.0 to 55.4%) in very low LC (Figure 4). It was 15.1% (CI95%11.6-19.4) for women and 7.0% (CI95% 4.9-9.9) for men. The value of this indicator by LC was 11.4% (CI95% 6.6-19.0) in high, 27.3% (CI95% 17.3-40.2) in intermediate, 20.6% (CI95% 10.5-36.4) low, and 26.9% (CI95% 9.7-55.9) in very low LC. The difference between the seroprevalence of high LC stratum when compared with the other three strata (intermediate, low and very low LC) was not significant (p=0.15). The Spearman correlation test evidenced independence between LCI and seroprevalence (r=0,11 and p=0,57). Figure 5 shows the heterogeneity in the distribution of seroprevalence by LC strata, according to the LCI.

Figure 3. A) Map of Census Tract (CT) of Feira de Santana by Living Conditions. B) Seroprevalence of Chikungunya virus by Sentinel Areas (SA).

Figure 4. Seroprevalence of CHIKV by Living Conditions strata in Feira de Santana. Bahia-Brazil, 2017.

Table 2. Population density, number of interviewed people, number of serosurvey participants, seroprevalence of chikungunya virus (CHIKV) and Premise Index (PI) by Living Condition (LC) and Sentinel Area (SA). Feira de Santana – Bahia, April to September, 2017.

LC	SA number	Population density [(1)] (inhab/km^2)	N Interviewed	% Female interviewed	N Serosurvey	Prev (%) CHIKV (CI95%)	Prev (%) CHIKV female (CI95%)	Prev(%) CHIKV male (CI95%)	PI [(2)]
**High**	6	5,026.4	559	59.0	50	10.0 (4.4-21.4)	9.1	11.8	1.73
	26	3,790.0	538	57.4	62	3.2 (0.01-11.0)	0.0	10.0	3.55
	23	4,665.5	892	64.6	60	16.7 (9.3-28.0)	17.5	15.0	2.64
	3	7,521.1	457	63.2	68	13.2 (7.1-23.3)	10.4	20.0	2.11
	5	6,297.9	624	60.6	41	9.8 (3.9-22.6)	11.5	6.7	2.42
	8	6,744.0	470	60.6	71	8.5 (3.9-17.2)	9.3	6.9	3.98
	**Mean / Total**	**5,674.2**	**3,540**	**60.9**	**352**	**[(3)]11.4 (6.6-19.0)**	**11.1 (5.5-21.2)**	**11.9 (7.4-18.4)**	**2.74**
	**Deff**	-	-	-	-	**1.07**	**1.11**	**0.40**	
**Intermediate**	16	5,133.0	274	66.4	47	34.0 (22.2-48.3)	36.1	27.3	1.22
	27	4,376.3	322	67.1	56	5.4 (1.8-14.6)	7.9	0.0	3.55
	9	5,669.1	293	67.2	55	12.7 (6.3-24.0)	16.2	5.6	1.39
	1	6,672.1	490	56.3	118	15.3 (9.9-22.8)	21.7	6.1	0.52
	14	6,120.8	607	58.8	82	36.6 (27.0-47.4)	39.7	29.2	2.89
	4	4,669.0	902	59.6	109	47.7 (38.6-57.0)	47.6	47.8	1.10
	24	6,801.0	802	56.1	65	32.3 (22.2-44.4)	36.8	25.9	1.05
	10	2,869.9	445	65.2	74	18.9 (11.6-29.3)	20.4	15.0	1.57
	7	4,648.9	657	60.3	53	11.3 (5.9-22.6)	8.3	16.7	1.02
	19	4,133.3	897	61.8	56	39.3 (27.6-52.4)	35.1	47.7	1.44
	2	7,780.1	758	58.7	71	8.5 (3.9-17.2)	10.9	4.0	1.68
	**Mean / Total**	**5,352.1**	**6,447**	**61.6**	**786**	**27.3 (17.3-40.2)**	**27.9 (18.5-39.7)**	**26.1 (14.1-43.5)**	**1.58**
	Deff	-	-	-	-	7.23	4.28	3.66	
**Low**	20	5,967.3	631	57.8	67	28.4 (19.0-40.1)	31.1	22.7	2.99
	28	4,895.3	552	73.7	82	8.5 (4.2-16.6)	10.8	0.0	0.78
	15	5,631.6	522	63.8	61	31.1 (20.9-43.6)	29.5	35.3	2.85
	29	5,865.3	476	59.9	51	2.0 (0.01-10.3)	0.0	6.7	4.50
	13	3,641.8	620	56.8	84	9.5 (4.9-17.7)	10.7	7.1	1.10
	22	4,463.9	877	56.1	70	18.6 (11.2-29.3)	15.9	23.1	1.44
	12	8,452.1	311	66.9	61	70.5 (58.11-80.4)	76.0	23.1	1.38
	21	5,109.9	404	67.3	96	8.3 (4.3-15.6)	9.1	6.7	2.19
	**Mean / Total**	**5,503.4**	**4,393**	**62.8**	**572**	**20.6 (10.5-36.4)**	**21.8 (10.2-40.7)**	**18.0 (10.1-29.9)**	**2.15**
	Deff	-	-	-	-	13.90	11.89	2.21	
**Very low**	17	4,537.9	479	63.0	56	55.4 (42.4-67.6)	55.6	55.0	2.41
	30	3,255.9	519	64.0	70	12.9 (6.9-22.7)	15.1	5.9	0.53
	25	6,206.7	393	58.0	36	11.1 (4.4-25.3)	12.5	8.3	1.40
	18	5,308.4	674	54.9	52	28.8 (18.3-42.3)	27.8	29.4	2.46

11	5,085.5	175	59.4	50	8.0 (3.2-18.8)	8.3	7.1	1.40
**Mean / Total**	**4,878.9**	**2,240**	**59.9**	**264**	**26.9 (9.7-55.9)**	**27.0 (10.4-54.0)**	**26.73 (8.1-60.3)**	**1.64**
Deff	-	-	-	-	7.49	5.06	2.62	
**Mean / Total**	**5,352.2**	**16,620**	**60.8**	**1,974**	**22.1 (16.7-28.6)**	**15.1 (11.6-19.4)**	**7.0 (4.9-9.9)**	**2,03**
Deff	-	-	-	-	9.11*	5.29	4.21	

1) Population Density estimated from data Brazilian Demographic Census, 2010.
2) Premise Index: % of all inspected premises which were found with at least one positive breeding site for *Aedes aegypti* larvae.
3) Difference not statistically significant when compared with the other living conditions strata.

* rho: 0.20 (CI95% 0.17 – 0.23)

Figure 5. Scatterplots for the relationship between CHIKV seroprevalence and Living Conditions Index of Sentinel Areas by Living Condition Strata in Feira de Santana. Bahia-Brazil, 2017.

The seroprevalence was higher in ≥40 years old (25.1%) than in < 40 years old (18.0%) and this difference was significant (p< 0.001) (data not shown). The seroprevalence ratios of chikungunya in the intermediate, low and very low LC strata were more than twice that of the high LC stratum. However, this ratio was statistically significant only in intermediate LC (Table 3).

Table 3. Seroprevalence and seroprevalence ratio of chikungunya by Living Condition (LC) strata. Feira Santana – Bahia, April to September, 2017.

**LC strata**	**Seroprevalence**		
	**%**	**Prevalence Ratio**	**95% confidence interval**
High	11.4	1	-
Intermediate	27.3	2.39	1.27-4.54
Low	20.6	1.81	0.87-3.77
Very low	26.9	2.36	0.96-5.81

There was negative correlation, no significant (r =-0.18; p= 0.34), between CHIKV seroprevalence and PI, whereas to population density it was positive and significant (r =0.24; p=0.02). Even removing the highest seroprevalence, the population density was still associated (r=0.24 and p=0.03).

Discussion

In this population-based study, the global CHIKV seroprevalence of the 30 SA of FSA was 22.1%, ranging from 2.0% to 70.5% among them. This seroprevalence was higher in women and in ≥ 40 years old. There was a weak positive correlation, but significant, between CHIKV seroprevalence and population density and also weak negative correlation, no significant, with PI. The seroprevalences of the LC strata did not show significant difference. However, the magnitude of seroprevalences of the SA included in each stratum, except in high LC, was very heterogeneous. The Spearman

correlation test evidenced independence between LCI and seroprevalence of SA for all LC strata.

The global seroprevalence in the SA of this study was 2.6 times lower than the 57% observed in the epicentre of the CHIKV epidemic in this same city, in 2015,[17] and it is noteworthy that only in two SA this indicator was higher than 50%. When we compare the results of this study with a seroprevalence survey of dengue, conducted with comparable methodology and a similar period after its re-emergence (about four years after disease introduction) in another Northeast Brazilian city (Salvador),[10] it was observed that the force of DENV transmission was more than three times higher (68.7% ranging from 16.2% to 97.6%) than CHIKV in FSA (22.1% ranging from 2.0% to 70.5%). It is important to highlight that although the seroprevalence of CHIKV was lower than observed in dengue transmission, chikungunya produces a high burden of chronic cases, mainly arthritis, resulting in a considerable negative impact on health and quality of life.[18] This disease can also contribute to the decompensation of associated comorbidities, that can even lead to death.[19,20] As observed in other studies,[17,21] we also found that older women are more affected by these infections. Besides, they are also those who present severe articular manifestations, which usually remain chronic for years.[22]

The inexistence of significant relation between CHIKV seroprevalence and the socioeconomic conditions observed is consistent with the findings of other authors[23]. However, conflicting results on this relationship are also found, as evidenced by a systematic review of the relationship between dengue and poverty.[24] A finding that stands out in our study is the heterogeneity of the magnitude of CHIKV seroprevalence in intra-urban spaces in the city of FSA, indicating that several factors, beyond living conditions, played a role in the occurrence of this infection, as the human population density that was correlated with CHIKV seroprevalence, although weakly. In turn, we did not find significant correlation with the PI, a fact that must have resulted from the time interval between the occurrence of infections in this study population and the entomological survey. In this meantime, vector control measures have been intensified, as informed by organs responsible by this task.[25]

In addition, the PI is an indicator that quantifies only the proportion of deposits with larvae in households and peridomiciliary houses. As already mentioned in the literature, it is not a good indicator of viral transmission, as it does not measure the

number of adult mosquitoes in the home environment[26]. Some authors also show that there is little evidence of an association between vector indices and dengue transmission that can be safely used to predict outbreaks.[27] On the other hand, it is known that clusters of inadequate houses in overcrowded places with lack of basic sanitation, water storage and accumulation of trash provide an ideal environment for vectors reproduction, mainly *Aedes aegypti*. Usually, these characteristics are common in neighborhoods with lower living conditions.

Although our study has found a weak relationship between the risk of being infected by CHIKV and population density, it is necessary to remember that, in general, the diseases transmitted by *Ae. aegypti* are modulated by population density.[28,29] We observed that the SA of highest CHIKV seroprevalence was a space with crowded residences and substandard slum housing, while the SA with the lowest seroprevalence was an area with mostly single story housing, with vacant lots between the residences. Specifically, the first one has higher population density than the second. This observation is consistent with knowledge about dengue, whose studies demonstrated a correlation between the prevalence of DENV infection and population density.[10,29,30] In turn, we also found SA with both relatively high (31.1% - SA 15) and low (2.0% - SA 29) seroprevalence levels with similar population density, 5,631 inhab/km^2 and 5,865 inhab/km^2, respectively.

These contrasts reveal that the study of the determinants of the occurrence of virus infections transmitted by mosquitoes of *Aedes* genus is very complex, especially in urban centres with co-circulation of three of these agents (DENV, CHIKV and ZIKV), as occur in FSA since 2015.[13] Possibly it is due to factors inherent to the receptivity of the physical environment[31] and socioeconomic conditions[32,33], beyond other factors involved, for example, the existence of competition between different agents for the vector, the possibility of co-infection in humans and mosquitoes[34] and the vector control measures adopted in each specific space[35].

In the case of FSA, we can hypothesize that the intensification of the vector control measures adopted after the emergence of CHIKV in the city was not universally implemented, but followed the logic of prioritizing the locations that initially produced the most symptomatic cases and that reached the knowledge of public health services. It may have influenced the virus circulation differently during the epidemics in those places that were the target of these actions.

The heterogeneity of the distribution of CHIKV infections can also be observed in intra-urban dissemination of the reported cases of the disease. In fact, the FSA epidemiological bulletins showed that, in 2014, when the CHIKV emerged in this city, two neighbourhoods (epicentres of the disease) concentrated 50% of the reported cases, while the other half was distributed in 74 different neighborhoods.[36] In 2015 and 2016, 11 and 16 neighbourhoods, respectively, accounted for 50% of cases, while the other were distributed in the rest of the 99 neighbourhoods that make up FSA.[25,37,38]

Our study has limitations due to the refusal to participate (22.1%) that can difficult the generalization of the results. As the refusal was mainly of men, it could have contributed to the encounter of greater seroprevalence among women. Besides that, we also did not investigate other factors that modulate the infections by arboviruses, as the availability of habitats for the vector or other environmental factors that influenced the abundance of *Aedes* in the houses; the seroprevalence of other arboviruses in the same time and space that can lead to competition between arboviruses. However, we understand that our investigation is relevant because it showed the transmission of CHIKV in a naive population, in a scenario of co-circulation with more two arboviruses that infect the same vector, in several urban spaces of the city less than three year before the emergence of this agent.

The great heterogeneity in attack rates of CHIKV in studied areas of FSA seems to be a characteristic of its dissemination, since there is a large variability in the seroprevalence levels on surveys available in the literature. These results indicate that the herd immunity levels and the maintenance of the infestations of the vector have been favoring the CHIKV circulation, either as endemic form or even in the form of new epidemics. In fact, between 2017 and 2019, cases of chikungunya were reported in FSA city with a relatively low incidence (ranging from 21 to 82 cases/100,000 inhabitants). However, in 2020 another epidemic broke out, which already reached an incidence of 610/100,000 inhabitants (up to 29th Epidemiological Week), that is, greater than in 2015, the year of greatest magnitude since the emergence of this agent in FSA.

In summary, the findings of this study reveal, as already demonstrated by other authors, that the factors that influence the greater or lesser risk of occurrence of urban arbovirus infections are highly complex at the ecological level. Even those that are considered as its most important determinants, such as the entomological situation, living conditions, population density and group immunity, they are not always clearly

identified from a statistical point of view. In other words, the results of the investigations point to controversies and uncertainties. Thus, it is necessary to seek the improvement of vector control technologies, which must be implemented universally in urban centres, as well as the improvements in living conditions, especially concerning basic sanitation infrastructure. In addition, initiatives aimed at the development of drugs against chikungunya are fundamental to reduce the occurrence of sequelae and deaths and, above all, a vaccine to prevent the occurrence of new infections since the vector control measures that have been applied for many decades have not been shown to be effective.

Article Summary Line

There is considerable heterogeneity in the occurrence of CHIKV infections in intraurban spaces being higher in more deprived areas, suggesting that the reduction of the incidence of this disease also depends on the improvement of the living conditions of the population.

Financial supporters: Medical Research Council/MRC; Coordenação de Aperfeiçoamento de Pessoal de Nível Superior/CAPES/ MoE Brazil; Secretaria de Vigilância em Saúde/ Fundo Nacional de Saúde/MoH Brazil.

Biograph Sketch

Dr. Teixeira is an infectious disease trained physician with PhD in Epidemiology. She is Titular Professor at Instituto de Saúde Coletiva, Federal University of Bahia, Brazil and her research interest is in the field of Infectious Diseases Epidemiology and Control, in special arboviruses.

Professor Skalinski is a nurse and trained in Field Epidemiology, MsC in Public Health and now she is a PhD student at Instituto de Saúde Coletiva, Federal University of Bahia, Brazil. Her interest is in Epidemiology Surveillance, Outbreak investigations and Epidemiology of Infectious Diseases.

References

1. World Health Organization. Chikungunya. WHO [Internet]. 2017 [cited 2019 Feb 28]; Available from: http://www.who.int/mediacentre/factsheets/fs327/en/

2. Pialoux G, Gaüzère BA, Jauréguiberry S, Strobel M. Chikungunya, an epidemic arbovirosis. Lancet Infect Dis. 2007;7(5):319–27.

3. Powers AM, Logue CH. Changing patterns of chikungunya virus: re-emergence of a zoonotic arbovirus. J Gen Virol. 2007;88:2363–77.

4. Riccardo F, Venturi G, Di Luca M, Del Manso M, Severini F, Andrianou X, et al. Secondary Autochthonous Outbreak of Chikungunya, Southern Italy, 2017. Emerg Infect Dis. 2019;25(11):2093–6.

5. Powers AM. Chikungunya. Clin Lab Med. 2010;30:209–19.

6. Nasci RS. Movement of Chikungunya virus into the Western Hemisphere. Emerg Infect Dis. 2014;20(8):1394–5.

7. Pan American Heatlh Organization. New Cases of Chikungunya in the Americas, per year and country.

8. Fritzell C, Rousset D, Adde A, Kazanji M, Van Kerkhove MD, Flamand C. Current challenges and implications for dengue, chikungunya and Zika seroprevalence studies worldwide: A scoping review. PLoS Negl Trop Dis. 2018;12(7):1–29.

9. Kikuti M, Cunha GM, Paploski IAD, Kasper AM, Silva MMO, Tavares AS, et al. Spatial distribution of dengue in a Brazilian Urban slum setting: Role of socioeconomic gradient in disease risk. PLoS Negl Trop Dis. 2015;9(7):1–18.

10. Teixeira MG, Barreto ML, Costa MCN, Ferreira LDA, Vasconcelos PF, Cairncross S. Dynamics of dengue virus circulation: a silent epidemic in a complex urban area. Trop Med Int Heal. 2002;7(9):757–762.

11. IBGE. Brasil em Síntese. Bahia. Feira de Santana. Panorama [Internet]. [cited 2019 Mar 8]. Available from: https://cidades.ibge.gov.br/brasil/ba/feira-de-santana/panorama

12. Teixeira MG, Andrade AMS, Costa MCN, Castro JSM, Oliveira FLS, Goes CSB, et al. East/central/South African genotype chikungunya virus, Brazil, 2014. Emerg Infect Dis. 2015;21(5):906–8.

13. Lourenço J, de Lima MM, Faria NR, Walker A, Kraemer MUG, Villabona-Arenas CJ, et al. Epidemiological and ecological determinants of Zika virus transmission in an urban setting. Elife. 2017;6(February 2015).

14. Paim JS, Silva LMV, Costa MCN, Prata PR, Lessa I. Desigualdades na situação de saúde do município de Salvador e relações com as condições de vida. 2003;30–9.

15. Pratt CM, Flusin O, Panella A, Tenebray B, Lanciotti R, Leparc-Goffart I. Evaluation of Commercially Available Serologic Diagnostic Tests for Chikungunya Virus. Emerg Infect Dis. 2014;20(12):2129–32.

16. Brasil.Ministério da Saúde. Secretaria de Vigilância em Saúde. Departamento de Vigilância das Doenças Transmissíveis. Levantamento Rápido de Índices para Aedes Aegypti - LIRAa - para vigilância entomológica do Aedes aegypti no Brasil. Brasília: Ministério da Saúde; 2012. 84 p.

17. Dias JP, Costa M da CN, Campos GS, Paixao ES, Natividade MS, Barreto FR, et

al. Seroprevalence of Chikungunya Virus in 2 Urban Areas of Brazil 1 Year after Emergence. Emerg Infect Dis [Internet]. 2018;24(4):617–24. Available from: https://wwwnc.cdc.gov/eid/article/24/4/pdfs/17-1370.pdf

18. Paixão ES, Rodrigues LC, Costa M da CN, Itaparica M, Barreto F, Gérardin P, et al. Chikungunya chronic disease: A systematic review and meta-analysis. Trans R Soc Trop Med Hyg. 2018;112(7):301–16.

19. Mavalankar D, Shastri P, Bandyopadhyay T, Parmar J, Ramani K V. Increased mortality rate associated with chikungunya epidemic, Almedabah, India. Emerg Infect Dis. 2008;14(3):412–5.

20. Brito CAA, Teixeira MG. Increased number of deaths during a chikungunya epidemic in Pernambuco, Brazil. Mem Inst Oswaldo Cruz. 2017;112(9):650–1.

21. Kama M, Aubry M, Naivalu T, Vanhomwegen J, Mariteragi-Helle T, Teissier A, et al. Sustained low-level transmission of zika and chikungunya viruses after emergence in the fiji islands. Emerg Infect Dis. 2019;25(8):1535–8.

22. Rodríguez-Morales AJ, Cardona-Ospina JA, Fernanda Urbano-Garzón S, Sebastian Hurtado-Zapata J. Prevalence of Post-Chikungunya Infection Chronic Inflammatory Arthritis: A Systematic Review and Meta-Analysis. Arthritis Care Res. 2016;68(12):1849–58.

23. McHale TC, Romero-Vivas CM, Fronterre C, Arango-Padilla P, Waterlow NR, Nix CD, et al. Spatiotemporal heterogeneity in the distribution of Chikungunya and Zika virus case incidences during their 2014 to 2016 epidemics in Barranquilla, Colombia. Int J Environ Res Public Health. 2019;16(10).

24. Mulligan K, Dixon J, Joanna Sinn C-L, Elliott SJ. Is dengue a disease of poverty? A systematic review. Pathog Glob Health. 2014;109(1):10–8.

25. Feira de Santana. Secretaria Municipal de Saúde. Vigilância Epidemiológica. Situação Epidemiológica dos casos de Chikungunya, Dengue, vírus Zika e Microcefalia. 2016.

26. Bowman LR, Runge-ranzinger S, Mccall PJ. Assessing the Relationship between Vector Indices and Dengue Transmission : A Systematic Review of the Evidence. 2014;8(5).

27. Bowman LR, Runge-Ranzinger S, McCall PJ. Assessing the Relationship between Vector Indices and Dengue Transmission: A Systematic Review of the Evidence. PLoS Negl Trop Dis. 2014;8(5).

28. Braga C, Luna CF, Martelli CMT, Souza WV de, Cordeiro MT, Alexander N, et al. Seroprevalence and risk factors for dengue infection in socio-economically distinct areas of Recife, Brazil. Acta Trop. 2010;

29. Teixeira MG, Barreto ML, Costa M da CN, Ferreira LDA, Morato V, Vasconcelos PF, et al. Exposure to the risk of dengue virus infection in an urban setting: Ecological versus individual heterogeneity. Dengue Bull. 2007;

30. Almeida AS, Medronho RA, Valencia LIO. Spatial analysis of dengue and the socioeconomic context of the city of Rio de Janeiro (Southeastern Brazil). Rev Saude Publica [Internet]. 2009;43(4):666–73. Available from: http://www.ncbi.nlm.nih.gov/pubmed/19649472

31. Arduino M de B, Mucci LF, Dos Santos LM, Soares MF de S. Importance of microenvironment to arbovirus vector distribution in an urban area, São Paulo, Brazil. Rev Soc Bras Med Trop. 2020;53(February):1–10.

32. Teixeira TRA, Medronho RA. Indicadores sócio-demográficos e a epidemia de dengue em 2002 no Estado do Rio de Janeiro, Brasil. Cad Saude Publica. 2008;24(9):2160–70.

33. Almeida MCM, Caiaffa WT, Assunção RM, Proietti FA. Spatial vulnerability to dengue in a Brazilian urban area during a 7-year surveillance. J Urban Heal. 2007;84(3):334–45.

34. Vogels CBF, Rückert C, Cavany SM, Perkins TA, Ebel GD, Grubaugh ND. Arbovirus coinfection and co-transmission: A neglected public health concern? PLoS Biol. 2019;17(1):1–16.

35. Abad-Franch F, Zamora-Perea E, Luz SLB. Mosquito-Disseminated Insecticide for Citywide Vector Control and Its Potential to Block Arbovirus Epidemics: Entomological Observations and Modeling Results from Amazonian Brazil. PLoS Med. 2017;14(1):1–22.

36. Prefeitura Municipal de Feira de Santana. Secretaria Municipal de Saúde. Vigilância Epidemiológica. Boletim da Febre do Chikungunya. 2014.

37. Feira de Santana. Secretaria Municipal de Saúde. Vigilância Epidemiológica. Situação Epidemiológica dos casos de Chikungunya, Dengue e Zika Vírus. 2015;27 jul (1).

38. Prefeitura Municipal de Feira de Santana. Secretaria do Governo. Regiões Administrativas [Internet]. [cited 2020 Jul 22]. Available from: http://www.feiradesantana.ba.gov.br/servicos.asp?id=2&link=segov/regioesadm.asp

Supporting Information Legends:

SI 1 – Strobe checklist: strobe.doc

SI 2 – Research database: database.xls

 Click here to access/download;Figure;figure 1.tif

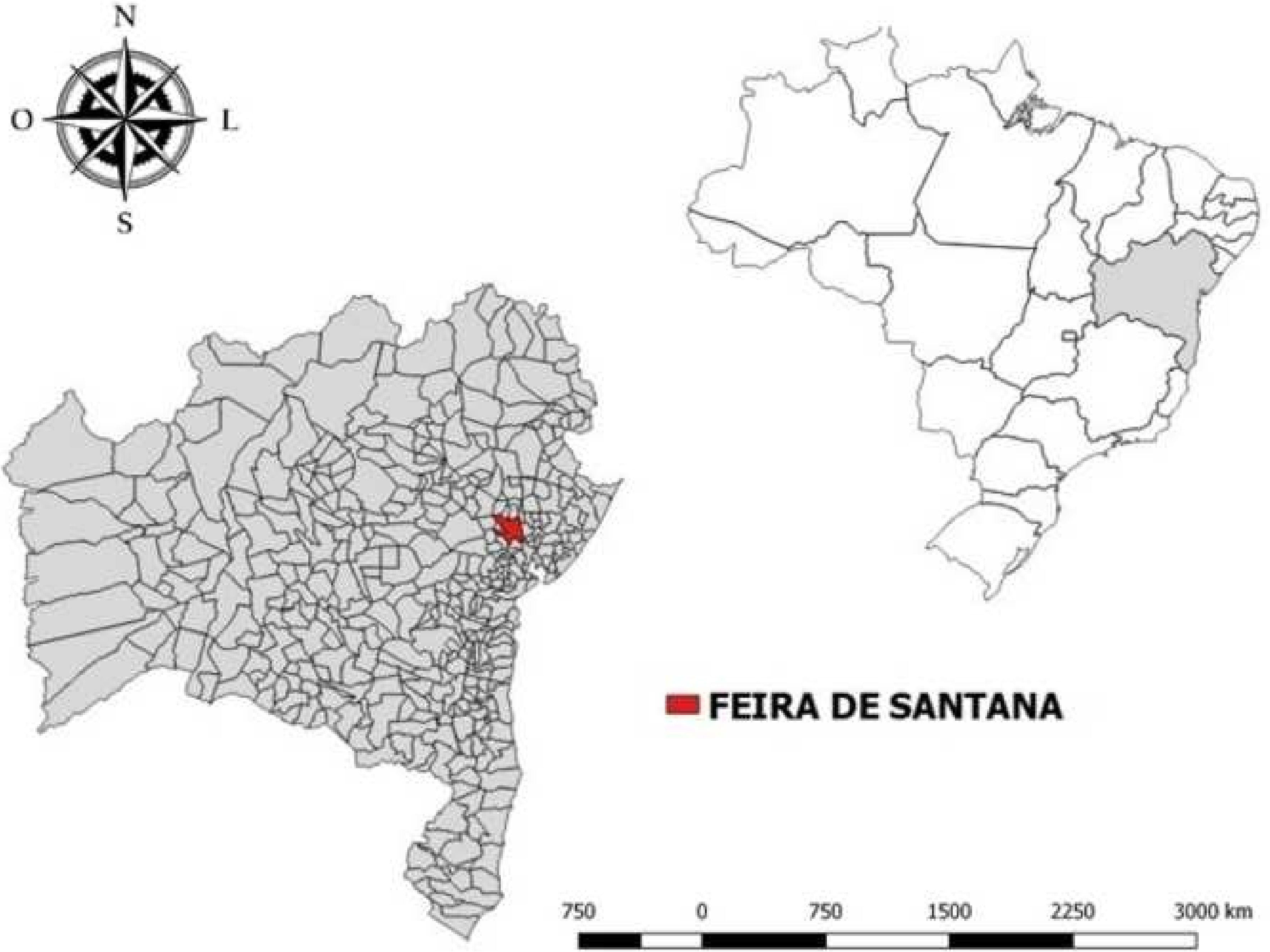

Figure2

Click here to access/download;Figure;Figure 2.docx

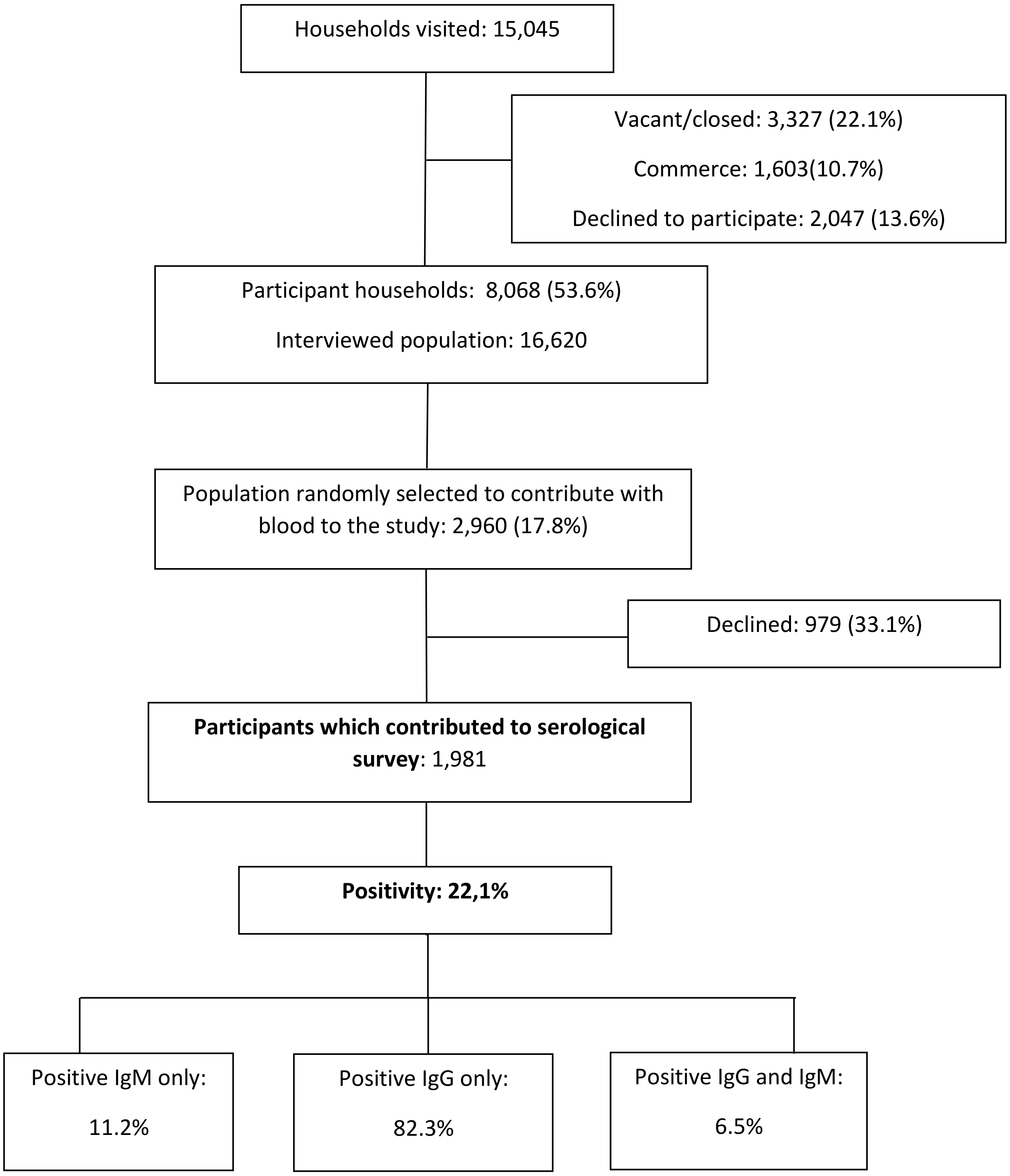

Figure3

Click here to access/download;Figure;figure 3.tif

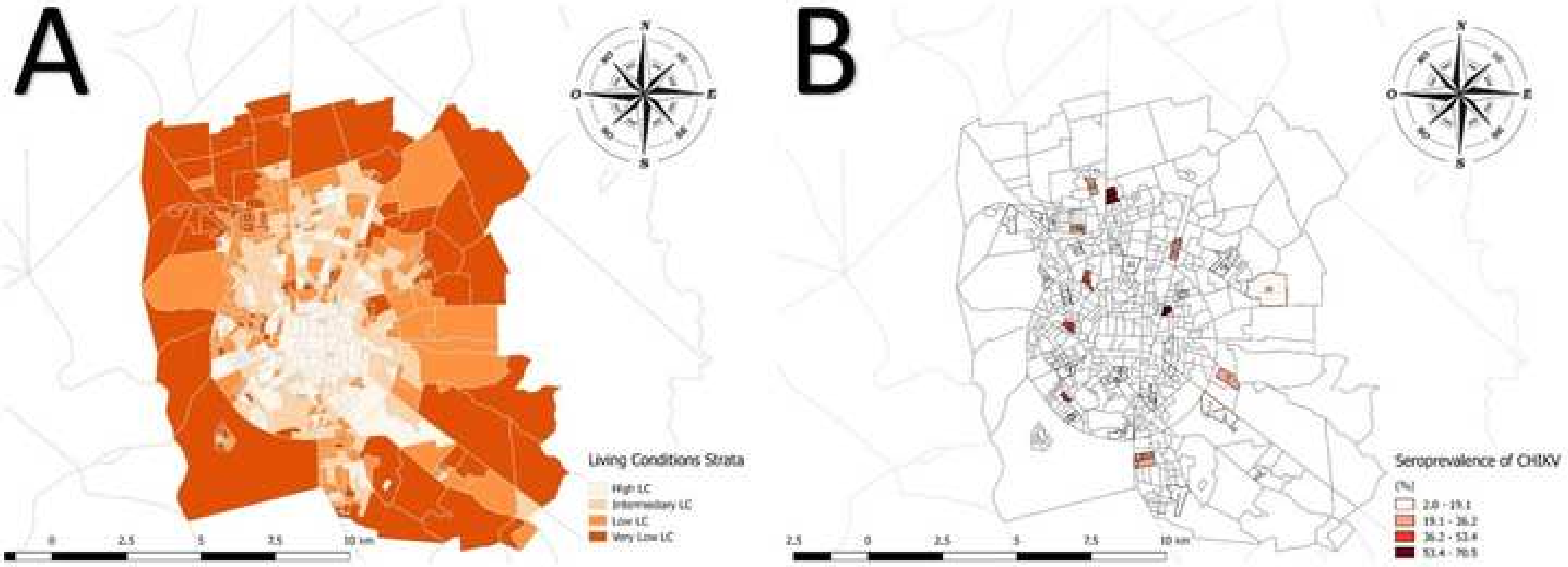

Figure4 Click here to access/download;Figure;Figure 4.doc

Figure 4. Seroprevalence of CHIKV by Living Conditions strata in Feira de Santana. Bahia-Brazil, 2017.

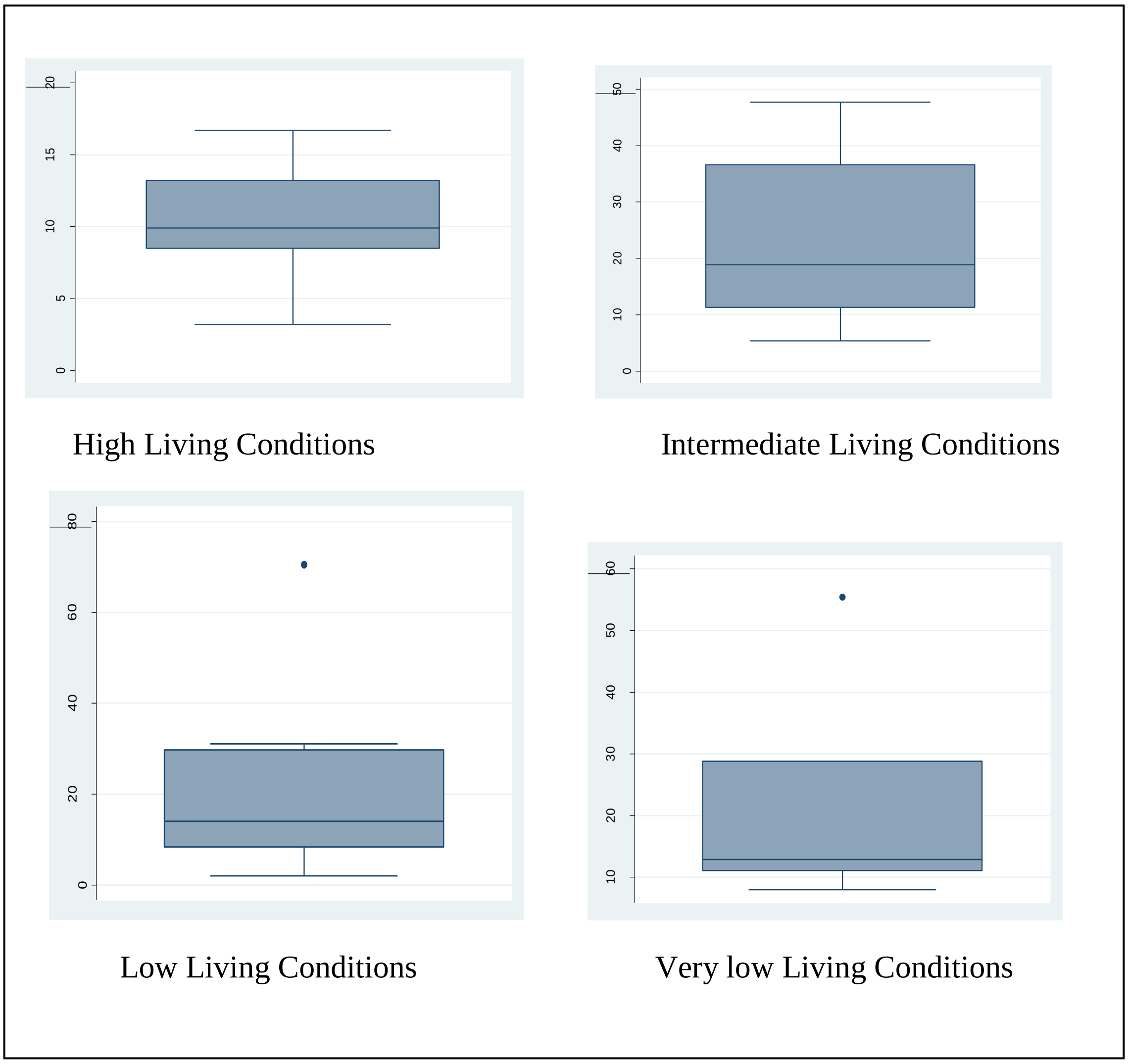

Figure5 Click here to access/download;Figure;Figure 5.doc

Figure 5. Scatterplots for the relationship between CHIKV seroprevalence and Living Conditions Index of Santinel Areas by Living Condition Strata in Feira de Santana. Bahia-Brazil, 2017.

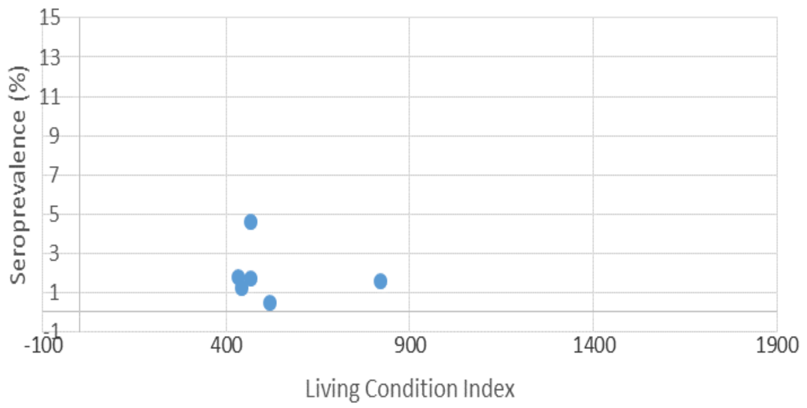

High Living Conditions (r= - 0.38; p= 0.46)

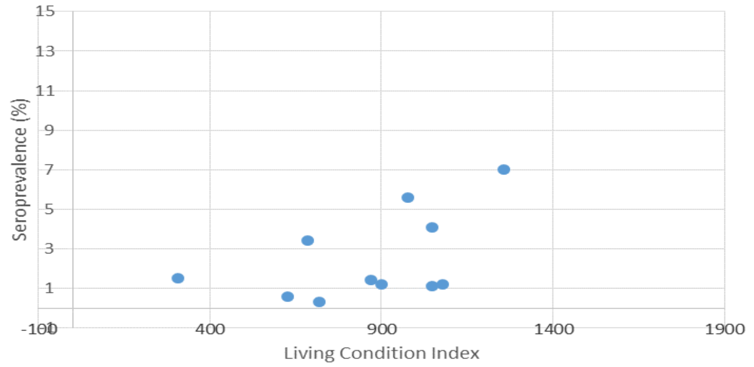

Intermediate Living Conditions (r=0.35; p= 0.30)

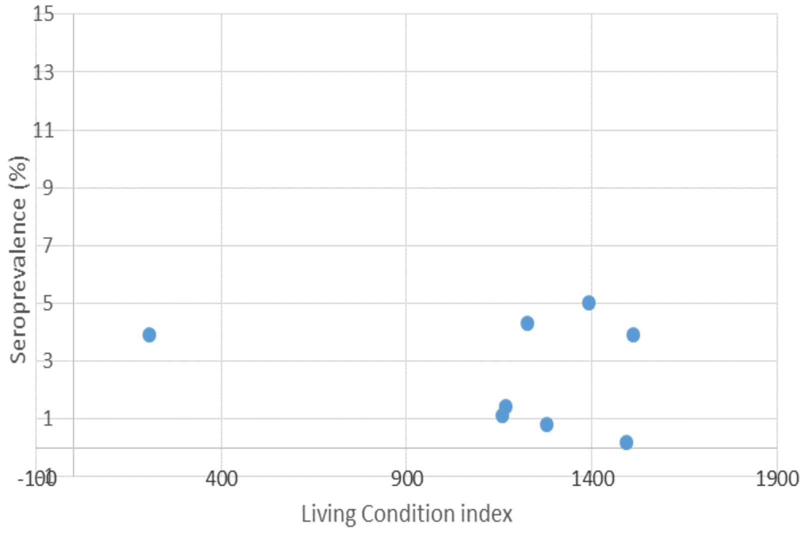

Low Living Conditions (r= - 0.04; p= 0.93)

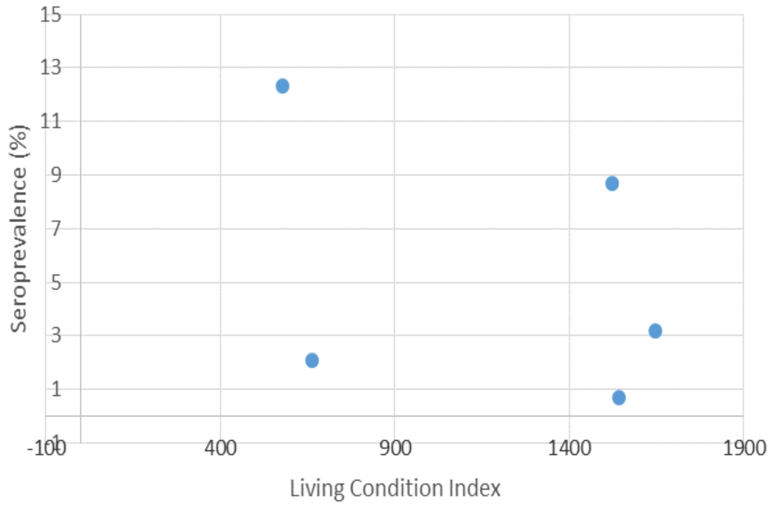

Very low Living Conditions (r= - 0.50; p= 0.39)

Click here to access/download
Supporting Information
strobe.doc

Click here to access/download
Supporting Information
database.xls

Reviewer's Responses to Questions

Reviewer #1:
The figure of CT (Fig.1B) and results of seroprevalence (Fig. 1C) should be put in section RESULTS instead of METHODS, since they are presenting the results of the study.
Thanks for the suggestion. We removed them from Methods and presented in the results section.

If the authors are to follow variable of ZI (Paim et al. 2003) and arranged the value of variables in ascending order (income, slum, crowding) and descending order (sanitation, education) and then to determine the LCI using the sum of each variable values, then the order of each variables should all go to the same direction of the living condition. If greater the sum value means higher the ILC, then orders for income, sanitation and education should be ascending, while crowding should be ordered descending, since authors excluded 'slum' from the variable list.
Thank you for alerting us. We replaced part of the paragraph, for better understanding. We explained how each one of the variables that make up the index was calculated by Paim (2003), and we added information about how the scores were ordered.

"To select the 30 SA we used the Living Conditions Index (LCI) as proxy of living conditions (LC), similar that proposed by Paim et al (2003).[14] For this, from the Demographic Census data of the year 2000, these authors used five indicators to the variables income (proportion of householders in permanent private households with average monthly income equal to or less than two minimum wages), slum (percentage of houses in "subnormal agglomerate" in relation to the total of households in the area), crowding (ratio between the average number of residents per household in the area and the average number of rooms serving dormitory in its area), sanitation (percentage of households with indoor plumbing connected to the global network of water supply) and education (proportion of persons 10 to 14 years old literate) for each Information Zone (IZ), unit of analysis employed in that study. Next, income, slum and subnormal agglomerate indicators were arranged in ascending order while education and sanitation were arranged in descending order considering the average value of each of them in the respective IZ. Then, each indicator received a consecutive value started by 1, according to the position assumed in this order and, after that, the values obtained by each one IZ were summed up thus obtaining a score representative of the living

conditions of the population of IZ. Subsequently, these scores were sorted in ascending order. Thus, higher scores corresponded to lower living conditions. Next, they were grouped in quartiles ZI corresponding to population segments classified in the following categories of living conditions: high, intermediate, low and very low.

Although for the present study all of these procedures have been adopted, as we used data from 2010 Census that did not have all information required for construction of the slum and crowding indicators, we had to exclude the first one and to build the last one as the "ratio inhabitants per room". The 30 SA were composed by a grouping of CT and they were selected considering socioeconomic and demographic criteria. Thus, the SA had greater uniformity within those in the same stratum of LC and greater heterogeneity between the strata."

Was any statistical correlation analysis been performed between ILC strata with seroprevalence rate of the CHIKV? (eg. Spearman correlation test).
We conducted the Spearman correlation test. It was not found statistical significance between LCI and seroprevalence (r=0,11 and p=0,57). These results were included in the paper.

Reviewer #2:

The design of the study, although not explicitly described, corresponds to a multistage survey based on sampling of clusters (sentinel areas; SA) from strata defined by an adaptation of a previously developed Living Conditions Index. Stratification was designed to minimize intra-stratum and maximize inter-strata heterogeneity of census tracts (CT); however, there is no mention to the procedure used to select clusters (SA) out of strata. Suggestion: Present a more precise and detailed description of the study design and sampling methodology.
Thanks for your suggestion. We have improved the explanation of how SA were selected.

"The Municipal Health Secretariat of Feira de Santana has a wide network of public health services distributed throughout the city's urban territory. As this survey is part of a platform of longitudinal clinical and epidemiological studies on dengue, Zika and chikungunya, we considered important that the population residing in the intra-urban spaces selected for these studies resided in the territories covered by Public Health Units. This way, in the course of the study, the research team would have access to information about the occurrence of cases of these arboviruses. Then, all SA should be

close to one Basic Health Unit (BHU) or Family Health Unit (FHU) that offered good quality care for the population. Professionals of Epidemiologic Surveillance of Feira de Santana/FSA city indicated the Basic Health Units (BHU) and the Family Health Units (FHU), which provided better quality care. They selected 46 of the 92 units located in urban FSA, which 14 were BHU. All 14 UBS were considered for the study because they performed complex care, and have health agents on their team. The other 16 SA were selected in CT where the FHU were not located in violent areas and offered good quality care. Health professionals who coordinated these units did this selection. As CT varied greatly in their population size, the SA had minimum 773 and maximum 2,442 people, according to IBGE data. Then, each SA had one, two or three CT with an average of 1,310 people residing in each SA, making a total of 40 CT."

Sample size calculation incorporated the "complex design" nature of the survey in the form of a design effect; however, there is no mention to the parameters used to derive it (i.e., intraclass correlation coefficient) or its resulting value. Suggestion: Include parameters used to estimate the design effect and present its actual value.
Thanks for warning us. We included in the manuscript the parameters used to estimate the design effect and, we recalculated the seroprevalence considering this parameters and presented the actual value of seroprevalence in table 2.

"The sample design was in a two-stage conglomerate, with the sample fraction of the 1st stage, the sentinel units (f1=1.0) that were self-represented, that is, they were all part of the study, and the sample fraction of the 2nd stage, selection of individuals (f2=f/f1) of 0.05, to compose the sample of each sentinel area, in order to maintain the simple random sampling fraction of 0.05 (f=f1*f2).
To calculate the sample size required for the study, the simple random sampling formula was used, with sample fraction f=n/N, considering the prevalence of the specific event of 50%; significance level of 5% and sampling error of 0.025. Thus, the sample size of 1,504 was obtained. This sample was expanded to 2,114 by assuming an effect around 1.42."

Finally, the data analysis section does not mention whether the complex design of the survey was incorporated into the point (prevalence) and uncertainty (confidence interval) estimation. Suggestion: Explain how complex design was incorporated in the process of estimation. If it was not the case (i.e., the analysis assumed simple random sampling), then the length of confidence intervals and type I error of statistical tests are underestimated.

After the procedures described in the previous question, we incorporated the survey effect in the analysis and presented the new seroprevalence estimates and respective CI95% for LC strata. We included this information in the manuscript.

Reviewer #1:
It is interesting to see the wide range of the prevalence rate for CHIKV in the city from 2% to 70% with mean only 20.8%. With the presence of SA with prevalence under 5% (2 SAs) and more than 50% (2 SAs), it is good to look with more detail, including the environment, availability of habitats and the dominant type of habitats, along with the entomology index and ILC, since both groups represented extreme polar of CHIKV exposures.
Unfortunately we can't proceed this analysis because we do not have data about habitats and environment. The data about Premise Index were extracted of the forms filled out by professionals of Entomological Surveillance System of the municipality, which do not have these informations. We included this important information as a limitation of study.

Also since CHIKV is transmitted by Aedes mosquitoes, as dengue viruses, that share the same ecosystem and habits, it will give more insight if we can compare side by side with dengue exposure in the same time periods (whether results of serology survey or record of dengue cases in each SA), whether the picture of both CHIKV and DENV similar in pattern or there is dominance of them that associated with some SAs.
Blood samples from this survey were collected to perform serology for dengue, Zika and Chikungunya, given that in Feira de Santana the three arboviruses circulate simultaneously. However, due to the cross reactions between IgG produced by ZIKV and DENV infections, since they are both flaviviruses, serological tests of good accuracy were not yet available. For this reason, it has not yet been possible to perform serological tests for these two viruses. As new diagnostic tests are emerging to more reliably identify IgG from Zika and dengue, such as DABA and BNITM, the same serum samples will be processed in the near future to ZIKV and DENV.

Reviewer #2:
Results are presented clearly, following a logical sequence, and in accordance with the analysis plan. These are some general comments/suggestions: 1) Use the decimal point consistently along the manuscript (see line 232 for an example) and 2) when presenting ranges place first the lowest and then the largest value (see lines 119 or 250).
Thanks. We corrected the decimal points and presentation of ranges.

More important, please comment on the reason(s) behind not presenting confidence intervals to prevalence estimates.
Thanks for your suggestion. We presented confidence intervals for seroprevalence estimates for LC strata. We add in the description of result and in Table 2.

In addition, figure 1 has poor resolution and in table 2 superscripts in parenthesis are not fully explained in the footnote.
Figures were constructed with better resolution.
We corrected the footnote of superscripts in table 2.

Reviewer #1:
The conclusion may biased if the authors did not consistent give mark value for each variable follow the order of the ILC, since the ILC value is the sum of all variables. Putting income and crowding in same order may affect the ILC since income may have positively affect ILC while crowding will affect it negatively.
We presented in the methods section a more complete description of the procedures to build the LCI for better understanding of the readers.

"To select the 30 SA we used the Living Conditions Index (LCI) as proxy of living conditions (LC), similar that proposed by Paim et al (2003).[14] For this, from the Demographic Census data of the year 2000, these authors used five indicators to the variables income (proportion of householders in permanent private households with average monthly income equal to or less than two minimum wages), slum (percentage of houses in "subnormal agglomerate" in relation to the total of households in the area), crowding (ratio between the average number of residents per household in the area and the average number of rooms serving dormitory in its area), sanitation (percentage of households with indoor plumbing connected to the global network of water supply) and education (proportion of persons 10 to 14 years old literate) for each Information Zone (IZ), unit of analysis employed in that study. Next, income, slum and subnormal agglomerate indicators were arranged in ascending order while education and sanitation were arranged in descending order considering the average value of each of them in the respective IZ. Then, each indicator received a consecutive value started by 1, according to the position assumed in this order and, after that, the values obtained by each one IZ were summed up thus obtaining a score representative of the living conditions of the population of IZ. Subsequently, these scores were sorted in ascending order. Thus, higher scores corresponded to lower living

conditions. Next, they were grouped in quartiles ZI corresponding to population segments classified in the following categories of living conditions: high, intermediate, low and very low.

Although for the present study all of these procedures have been adopted, as we used data from 2010 Census that did not have all information required for construction of the slum and crowding indicators, we had to exclude the first one and to build the last one as the "ratio inhabitants per room". The 30 SA were composed by a grouping of CT and they were selected considering socioeconomic and demographic criteria. Thus, the SA had greater uniformity within those in the same stratum of LC and greater heterogeneity between the strata."

Low seroprevalence should mean that the area has low exposure of CHIKV, whether it was due to scarce of virus or inability of viruses to transmit due to environment that do not support for vector abundance. No clear analysis on the prevalence rate and entomological indicators. Of course, the low herd immunity will increase the vulnerability for an outbreak in the future. To say that the vulnerability will to be an endemic status, please check the data for at least last three years, if it show that there some cases of CHIKV at each of the year, than it is already endemic, if not than future outbreak and the vector availability can move the area to endemic state.
Thanks for your comment. We improved the discussion data about entomological indicator and data about last years.

"These contrasts reveal that the study of the determinants of the occurrence of virus infections transmitted by mosquitoes of *Aedes* genus is very complex, especially in urban centers with co-circulation of three of these agents (DENV, CHIKV and ZIKV), as occur in FSA since 2015.[13] Possibly it is due to factors inherent to the receptivity of the physical environment[31] and socioeconomic conditions[32,33], beyond other factors involved, for example, the existence of competition between different agents for the vector, the possibility of co-infection in humans and mosquitoes[34] and the vector control measures adopted in each specific space[35].
In the case of FSA, we can hypothesize that the intensification of the vector control measures adopted after the emergence of CHIKV in the city were not universally implemented, but followed the logic of prioritizing the locations that initially produced the most symptomatic cases and that reached the knowledge of public health services. It may have influenced differently the virus circulation during the epidemics in those places that were the target of these actions.
The heterogeneity of the distribution of CHIKV infections can also be observed in intra-urban dissemination of the reported cases of the disease. In

fact, the FSA epidemiological bulletins showed that, in 2014, when the CHIKV emerged in this city, two neighborhoods (epicenters of the disease) concentrated 50% of the reported cases, while the other half was distributed in 74 different neighborhoods.[36] In 2015 and 2016, 11 and 16 neighborhoods, respectively, accounted for 50% of cases, while the other were distributed in the rest of the 99 neighborhoods that make up FSA.[25,37,38]"

Authors concluded that risk of infected by CHIKV strongly modulated by population density but did not show any analysis to correlate the population density of SA with the seroprevalence.
Sorry for the mistake. The correlation result found statistical significance, but it was not strong. We corrected it in the results and conclusion.

Reviewer #2:
The conclusions of the study (a low average seroprevalence, an inverse relation of seroprevalence and LC, and the existence of high heterogeneity within strata of LC) are supported by the data; however, I recommend to focus on and develop further the discussion of the main finding of the study in relation to the CHKV seroprevalence estimate (lines 331-333).
In relation to the observed high heterogeneity of seroprevalence, I also suggest to deep into the discussion of potential explanations. I am not certain that population density is a factor beyond LC, as this index included crowding. Also, in the study, population density was weakly correlated to seroprevalence and did not seem to explain heterogeneity either (i.e., SA 15 had seroprevalence of 31% and a population density of 5,632 as compared to SA 29 that had seroprevalence of 2% and a population density of 5,865).
Thanks for your suggestion. We improved the discussion about the heterogeneity of seroprevalence and commented about population density.

"The great heterogeneity in attack rates of CHIKV in studied areas of FSA seems to be a characteristic of its dissemination, since there is a large variability in the seroprevalence levels on surveys available in literature. These results indicate that the herd immunity levels and the maintenance of the infestations of the vector have been favoring the CHIKV circulation, either as endemic form or even in the form of new epidemics. In fact, between 2017 and 2019, cases of chikungunya were reported in FSA city with a relatively low incidence (ranging from 21 to 82 cases/100,000 inhabitants). However, in 2020 another epidemic broke out, which already reached an incidence of 610/100,000 inhabitants (up to 29th Epidemiological Week), that is, greater than in 2015, the year of greatest magnitude since the emergence of this agent in FSA."

“In summary, the findings of this study reveal, as already demonstrated by other authors, that the factors that influence the greater or lesser risk of occurrence of urban arbovirus infections are highly complex at the ecological level. Even those that are considered as its most important determinants, such as the entomological situation, living conditions, population density and group immunity, they are not always clearly identified from a statistical point of view. In other words, the results of the investigations point to controversies and uncertainties. Thus, it is necessary to seek the improvement of vector control technologies, which must be implemented universally in urban centers, as well as the improvements in living conditions, especially with regard to basic sanitation infrastructure.”

“Although our study have found a weak relationship between the risk of being infected by CHIKV and population density, it is necessary to remember that, in general, the diseases transmitted by *Ae. aegypti* are modulated by population density.[28,29] We observed that the SA of highest CHIKV seroprevalence was a space with crowded residences and substandard slum housing, while the SA with lowest seroprevalence was an area with mostly single story housing, with vacant lots between the residences. Specifically, the first one has higher population density than the second. This observation is consistent with knowledge about dengue, whose studies demonstrated a correlation between the prevalence of DENV infection and population density.[10,29,30] In turn, we also found SA with both relatively high (31.1% - SA 15) and low (2.0% - SA 29) seroprevalence levels with similar population density, 5,631 inhab/km^2 and 5,865 inhab/km^2, respectively.”

Please check line 348 for double negative.
Thanks. We corrected it.

The authors declare that their study has “limitations” (line 367), however, they only present one in relation to refusals without further development of the implication(s) of such bias (i.e., selection bias) to the results or their generalizability.
We detailed more information about limitations.

“Our study has limitations due to the refusal to participate (22.1%) that can difficult the generalization of the results. As the refusal was mainly of men, it could have contributed to the encounter of greater seroprevalence among women. Besides that, we also did not investigate other factors that modulate the infections by arboviruses, as the availability of habitats for the vector or other environmental factors that influenced the abundance of *Aedes* in the houses; the seroprevalence of other arboviruses in the same time and space

that can lead to competition between arboviruses. However, we understand that our investigation is relevant because it showed the transmission of CHIKV in a naive population, in a scenario of co-circulation with more two arboviruses that infect the same vector, in several intra urban spaces of the city less than three year before the emergence of this agent.”

Reviewer #1:
All figures very poor in resolution and need to be re-draw to increase the clearness and match the required resolution.
We re-drew the figures with better resolution.

Since the title of the manuscript sounds to explore the association of CHIKV seroprevalence with the living condition, it will helpful to present a scatter plot data of each SA versus ILC strata (or even ILC original value, the sum value of all variables). It also good to present the comparison of seroprevalence rate of each ILC strata in boxplot.
Thanks for your suggestion. We elaborated scatter plots about seroprevalence and LCI (sum of all variables) for the four LC strata. Following the other suggestion, we elaborated box plot graphs about seroprevalence rates by LC strata. We add the graphs in results section.

Authors did not consistent in positioning ‘mean’ for each LCI strata. In table 1 it was put at the top of the column while in table 2 was at the bottom of the column for each stratum.

We corrected table 2.

Reviewer #1:
Line 67: Prefer to expand LC to be living condition
We expanded it.

Line 84: Better to mention completely the name of the city in Summary.
We expanded it.

Line 119: better to re-write the number as “0.4% to over 75%”, instead of “over 75% to 0.4%”
We corrected it.

Line 146: write the complete name of IBGE or make a citation to reference #14.
We wrote the complete name of IBGE.

Line 169-170: How this range of population size of each SA was determined?
Thanks for your question. We noticed a mistake in the sentence: "As CT varied greatly in their population size, it was necessary that each SA would have minimum 773 and maximum 2,442 people." The correct is: "As CT varied greatly in their population size, the SA had minimum 773 and maximum 2,442 people, according to IBGE data."

Line 170: …each SA had one, two or three CT (Figure 1C). This is not clear in the Figure.
Thanks for warning about it. The Figure 1C did not present this information. We removed figure 1C from Methods section and add in results section, where we present the seroprevalence.

Line 308-312: Was any statistical correlation analysis been performed between ILC strata with seroprevalence rate of the CHIKV? (eg. Spearman correlation test).
Thanks for your suggestion. We performed the Spearman correlation test and did not find any significant statistical correlation. We add this information in the results section.

Line 359-360: Please show the statistical analysis using the study data to support the claim.
Thanks for your suggestion. We removed this claim from discussion and commented that the correlation was weak. Statistical data about this correlation were showed in results section.

"There was negative correlation, no significant ($r = -0.18$; $p = 0.34$), between CHIKV seroprevalence and PI, whereas to population density it was positive and significant ($r = 0.24$; $p = 0.02$). Even removing the highest seroprevalence, the population density was still associated ($r = 0.24$ and $p = 0.03$)."

"A finding that stands out in our study is the heterogeneity of the magnitude of CHIKV seroprevalence in intra-urban spaces in the city of FSA, indicating that several factors, beyond living conditions, played a role in the occurrence of this infection, as example the human population density that was correlated with CHIKV seroprevalence, although weakly."

Line 369-370: Please back up this statement with statistical test as mentioned above (line 308-312 and Line 359-360).
You are right. We included this information in the indicated lines.

Reviewer #2:
Teixeira et al. designed and conducted a population-based, chikungunya virus (CHKV) seroprevalence study in Feira de Santana, Bahia-Brazil. The design of the study and the execution of both field and laboratory procedures in such large sample size are some of the main strengths of the survey, although there are missing details in the description of the methods that should be addressed before publication, particularly related to sampling and incorporation of its complexity in the analysis. Perhaps the main weakness of the study is not related to issues of design or execution but the description of the methodology and the discussion of findings. I strongly encourage the authors to accomplish a more exhaustive review of alternative explanations to the observed high intraurban seroprevalence heterogeneity.
Once again we thank the reviewers for the valuable suggestions, which have been fully accepted throughout the manuscript.

Data Requirements:

Please note that, as a condition of publication, PLOS' data policy requires that you make available all data used to draw the conclusions outlined in your manuscript. Data must be deposited in an appropriate repository, included within the body of the manuscript, or uploaded as supporting information. This includes all numerical values that were used to generate graphs, histograms etc.. For an example see here: http://www.plosbiology.org/article/info%3Adoi%2F10.1371%2Fjournal.pbio.1001908#s5.
We attached the the database among other documents for submission.

Click here to access/download
Revised Article with Changes Highlighted
plos0308_versaosuja.doc
