# Supplementary material for: Seroprevalence of Chikungunya virus and living conditions in Feira de Santana, Bahia-Brazil
Source: PLoS Negl Trop Dis. 2021 Apr 20;15(4):e0009289. doi: 10.1371/journal.pntd.0009289 (PMC8087031; doi:10.1371/journal.pntd.0009289)
Supplement: Attachment — Submitted filename: resposta pntd.docx [file pntd.0009289.s003.docx]

**Reviewer's Responses to Questions**

Reviewer #1:

The figure of CT (Fig.1B) and results of seroprevalence (Fig. 1C) should be put in section RESULTS instead of METHODS, since they are presenting the results of the study.

Thanks for the suggestion. We removed them from Methods and presented in the results section.

If the authors are to follow variable of ZI (Paim et al. 2003) and arranged the value of variables in ascending order (income, slum, crowding) and descending order (sanitation, education) and then to determine the LCI using the sum of each variable values, then the order of each variables should all go to the same direction of the living condition. If greater the sum value means higher the ILC, then orders for income, sanitation and education should be ascending, while crowding should be ordered descending, since authors excluded ‘slum’ from the variable list.

Thank you for alerting us. We replaced part of the paragraph, for better understanding. We explained how each one of the variables that make up the index was calculated by Paim (2003), and we added information about how the scores were ordered.

“To select the 30 SA we used the Living Conditions Index (LCI) as proxy of living conditions (LC), similar that proposed by Paim et al (2003).^14^ For this, from the Demographic Census data of the year 2000, these authors used five indicators to the variables income (proportion of householders in permanent private households with average monthly income equal to or less than two minimum wages), slum (percentage of houses in “subnormal agglomerate” in relation to the total of households in the area), crowding (ratio between the average number of residents per household in the area and the average number of rooms serving dormitory in its area), sanitation (percentage of households with indoor plumbing connected to the global network of water supply) and education (proportion of persons 10 to 14 years old literate) for each Information Zone (IZ), unit of analysis employed in that study.  Next, income, slum and subnormal agglomerate indicators were arranged in ascending order while education and sanitation were arranged in descending order considering the average value of each of them in the respective IZ. Then, each indicator received a consecutive value started by 1, according to the position assumed in this order and, after that, the values obtained by each one IZ were summed up thus obtaining a score representative of the living conditions of the population of IZ. Subsequently, these scores were sorted in ascending order. Thus, higher scores corresponded to lower living conditions. Next, they were grouped in quartiles ZI corresponding to population segments classified in the following categories of living conditions: high, intermediate, low and very low.

Although for the present study all of these procedures have been adopted, as we used data from 2010 Census that did not have all information required for construction of the slum and crowding indicators, we had to exclude the first one and to build the last one as the “ratio inhabitants per room”. The 30 SA were composed by a grouping of CT and they were selected considering socioeconomic and demographic criteria. Thus, the SA had greater uniformity within those in the same stratum of LC and greater heterogeneity between the strata.”

Was any statistical correlation analysis been performed between ILC strata with seroprevalence rate of the CHIKV? (eg. Spearman correlation test).

We conducted the Spearman correlation test. It was not found statistical significance between LCI and seroprevalence (r=0,11 and p=0,57). These results were included in the paper.

Reviewer #2:

The design of the study, although not explicitly described, corresponds to a multistage survey based on sampling of clusters (sentinel areas; SA) from strata defined by an adaptation of a previously developed Living Conditions Index. Stratification was designed to minimize intra-stratum and maximize inter-strata heterogeneity of census tracts (CT); however, there is no mention to the procedure used to select clusters (SA) out of strata. Suggestion: Present a more precise and detailed description of the study design and sampling methodology.

Thanks for your suggestion. We have improved the explanation of how SA were selected.

“The Municipal Health Secretariat of Feira de Santana has a wide network of public health services distributed throughout the city's urban territory. As this survey is part of a platform of longitudinal clinical and epidemiological studies on dengue, Zika and chikungunya, we considered important that the population residing in the intra-urban spaces selected for these studies resided in the territories covered by Public Health Units. This way, in the course of the study, the research team would have access to information about the occurrence of cases of these arboviruses. Then, all SA should be close to one Basic Health Unit (BHU) or Family Health Unit (FHU) that offered good quality care for the population.  Professionals of Epidemiologic Surveillance of Feira de Santana/FSA city indicated the Basic Health Units (BHU) and the Family Health Units (FHU), which provided better quality care. They selected 46 of the 92 units located in urban FSA, which 14 were BHU. All 14 UBS were considered for the study because they performed complex care, and have health agents on their team. The other 16 SA were selected in CT where the FHU were not located in violent areas and offered good quality care. Health professionals who coordinated these units did this selection. As CT varied greatly in their population size, the SA had minimum 773 and maximum 2,442 people, according to IBGE data. Then, each SA had one, two or three CT with an average of 1,310 people residing in each SA, making a total of 40 CT.”

Sample size calculation incorporated the “complex design” nature of the survey in the form of a design effect; however, there is no mention to the parameters used to derive it (i.e., intraclass correlation coefficient) or its resulting value. Suggestion: Include parameters used to estimate the design effect and present its actual value.

Thanks for warning us. We included in the manuscript the parameters used to estimate the design effect and, we recalculated the seroprevalence considering this parameters and presented the actual value of seroprevalence in table 2.

“The sample design was in a two-stage conglomerate, with the sample fraction of the 1st stage, the sentinel units (f1=1.0) that were self-represented, that is, they were all part of the study, and the sample fraction of the 2nd stage, selection of individuals (f2=f/f1) of 0.05, to compose the sample of each sentinel area, in order to maintain the simple random sampling fraction of 0.05 (f=f1*f2).

To calculate the sample size required for the study, the simple random sampling formula was used, with sample fraction f=n/N, considering the prevalence of the specific event of 50%; significance level of 5% and sampling error of 0.025. Thus, the sample size of 1,504 was obtained. This sample was expanded to 2,114 by assuming an effect around 1.42.”

Finally, the data analysis section does not mention whether the complex design of the survey was incorporated into the point (prevalence) and uncertainty (confidence interval) estimation. Suggestion: Explain how complex design was incorporated in the process of estimation. If it was not the case (i.e., the analysis assumed simple random sampling), then the length of confidence intervals and type I error of statistical tests are underestimated.
After the procedures described in the previous question, we incorporated the survey effect in the analysis and presented the new seroprevalence estimates and respective CI95% for LC strata. We included this information in the manuscript.

Reviewer #1:

It is interesting to see the wide range of the prevalence rate for CHIKV in the city from 2% to 70% with mean only 20.8%. With the presence of SA with prevalence under 5% (2 SAs) and more than 50% (2 SAs), it is good to look with more detail, including the environment, availability of habitats and the dominant type of habitats, along with the entomology index and ILC, since both groups represented extreme polar of CHIKV exposures.

Unfortunately we can’t proceed this analysis because we do not have data about habitats and environment. The data about Premise Index were extracted of the forms filled out by professionals of Entomological Surveillance System of the municipality, which do not have these informations. We included this important information as a limitation of study.

Also since CHIKV is transmitted by Aedes mosquitoes, as dengue viruses, that share the same ecosystem and habits, it will give more insight if we can compare side by side with dengue exposure in the same time periods (whether results of serology survey or record of dengue cases in each SA), whether the picture of both CHIKV and DENV similar in pattern or there is dominance of them that associated with some SAs.

Blood samples from this survey were collected to perform serology for dengue, Zika and Chikungunya, given that in Feira de Santana the three arboviruses circulate simultaneously. However, due to the cross reactions between IgG produced by ZIKV and DENV infections, since they are both flaviviruses, serological tests of good accuracy were not yet available. For this reason, it has not yet been possible to perform serological tests for these two viruses. As new diagnostic tests are emerging to more reliably identify IgG from Zika and dengue, such as DABA and BNITM, the same serum samples will be processed in the near future to ZIKV and DENV.

Reviewer #2:

Results are presented clearly, following a logical sequence, and in accordance with the analysis plan. These are some general comments/suggestions: 1) Use the decimal point consistently along the manuscript (see line 232 for an example) and 2) when presenting ranges place first the lowest and then the largest value (see lines 119 or 250).

Thanks. We corrected the decimal points and presentation of ranges.

More important, please comment on the reason(s) behind not presenting confidence intervals to prevalence estimates.

Thanks for your suggestion. We presented confidence intervals for seroprevalence estimates for LC strata. We add in the description of result and in Table 2.

In addition, figure 1 has poor resolution and in table 2 superscripts in parenthesis are not fully explained in the footnote.

Figures were constructed with better resolution.

We corrected the footnote of superscripts in table 2.

Reviewer #1:

The conclusion may biased if the authors did not consistent give mark value for each variable follow the order of the ILC, since the ILC value is the sum of all variables. Putting income and crowding in same order may affect the ILC since income may have positively affect ILC while crowding will affect it negatively.

We presented in the methods section a more complete description of the procedures to build the LCI for better understanding of the readers.

“To select the 30 SA we used the Living Conditions Index (LCI) as proxy of living conditions (LC), similar that proposed by Paim et al (2003).^14^ For this, from the Demographic Census data of the year 2000, these authors used five indicators to the variables income (proportion of householders in permanent private households with average monthly income equal to or less than two minimum wages), slum (percentage of houses in “subnormal agglomerate” in relation to the total of households in the area), crowding (ratio between the average number of residents per household in the area and the average number of rooms serving dormitory in its area), sanitation (percentage of households with indoor plumbing connected to the global network of water supply) and education (proportion of persons 10 to 14 years old literate) for each Information Zone (IZ), unit of analysis employed in that study.  Next, income, slum and subnormal agglomerate indicators were arranged in ascending order while education and sanitation were arranged in descending order considering the average value of each of them in the respective IZ. Then, each indicator received a consecutive value started by 1, according to the position assumed in this order and, after that, the values obtained by each one IZ were summed up thus obtaining a score representative of the living conditions of the population of IZ. Subsequently, these scores were sorted in ascending order. Thus, higher scores corresponded to lower living conditions. Next, they were grouped in quartiles ZI corresponding to population segments classified in the following categories of living conditions: high, intermediate, low and very low.

Although for the present study all of these procedures have been adopted, as we used data from 2010 Census that did not have all information required for construction of the slum and crowding indicators, we had to exclude the first one and to build the last one as the “ratio inhabitants per room”. The 30 SA were composed by a grouping of CT and they were selected considering socioeconomic and demographic criteria. Thus, the SA had greater uniformity within those in the same stratum of LC and greater heterogeneity between the strata.”

Low seroprevalence should mean that the area has low exposure of CHIKV, whether it was due to scarce of virus or inability of viruses to transmit due to environment that do not support for vector abundance. No clear analysis on the prevalence rate and entomological indicators. Of course, the low herd immunity will increase the vulnerability for an outbreak in the future. To say that the vulnerability will to be an endemic status, please check the data for at least last three years, if it show that there some cases of CHIKV at each of the year, than it is already endemic, if not than future outbreak and the vector availability can move the area to endemic state.

Thanks for your comment. We improved the discussion data about entomological indicator and data about last years.

“These contrasts reveal that the study of the determinants of the occurrence of virus infections transmitted by mosquitoes of *Aedes* genus is very complex, especially in urban centers with co-circulation of three of these agents (DENV, CHIKV and ZIKV), as occur in FSA since 2015.^13^ Possibly it is due to factors inherent to the receptivity of the physical environment^31^ and socioeconomic conditions^32,33^, beyond other factors involved, for example, the existence of competition between different agents for the vector, the possibility of co-infection in humans and mosquitoes^34^ and the vector control measures adopted in each specific space^35^.

In the case of FSA, we can hypothesize that the intensification of the vector control measures adopted after the emergence of CHIKV in the city were not universally implemented, but followed the logic of prioritizing the locations that initially produced the most symptomatic cases and that reached the knowledge of public health services. It may have influenced differently the virus circulation during the epidemics in those places that were the target of these actions.

The heterogeneity of the distribution of CHIKV infections can also be observed in intra-urban dissemination of the reported cases of the disease. In fact, the FSA epidemiological bulletins showed that, in 2014, when the CHIKV emerged in this city, two neighborhoods (epicenters of the disease) concentrated 50% of the reported cases, while the other half was distributed in 74 different neighborhoods.^36^ In 2015 and 2016, 11 and 16 neighborhoods, respectively, accounted for 50% of cases, while the other were distributed in the rest of the 99 neighborhoods that make up FSA.^25,37,38”^

Authors concluded that risk of infected by CHIKV strongly modulated by population density but did not show any analysis to correlate the population density of SA with the seroprevalence.

Sorry for the mistake. The correlation result found statistical significance, but it was not strong. We corrected it in the results and conclusion.

Reviewer #2:

The conclusions of the study (a low average seroprevalence, an inverse relation of seroprevalence and LC, and the existence of high heterogeneity within strata of LC) are supported by the data; however, I recommend to focus on and develop further the discussion of the main finding of the study in relation to the CHKV seroprevalence estimate (lines 331-333).

In relation to the observed high heterogeneity of seroprevalence, I also suggest to deep into the discussion of potential explanations. I am not certain that population density is a factor beyond LC, as this index included crowding. Also, in the study, population density was weakly correlated to seroprevalence and did not seem to explain heterogeneity either (i.e., SA 15 had seroprevalence of 31% and a population density of 5,632 as compared to SA 29 that had seroprevalence of 2% and a population density of 5,865).

Thanks for your suggestion. We improved the discussion about the heterogeneity of seroprevalence and commented about population density.

“The great heterogeneity in attack rates of CHIKV in studied areas of FSA seems to be a characteristic of its dissemination, since there is a large variability in the seroprevalence levels on surveys available in literature. These results indicate that the herd immunity levels and the maintenance of the infestations of the vector have been favoring the CHIKV circulation, either as endemic form or even in the form of new epidemics. In fact, between 2017 and 2019, cases of chikungunya were reported in FSA city with a relatively low incidence (ranging from 21 to 82 cases/100,000 inhabitants). However, in 2020 another epidemic broke out, which already reached an incidence of 610/100,000 inhabitants (up to 29^th^ Epidemiological Week), that is, greater than in 2015, the year of greatest magnitude since the emergence of this agent in FSA.”

“In summary, the findings of this study reveal, as already demonstrated by other authors, that the factors that influence the greater or lesser risk of occurrence of urban arbovirus infections are highly complex at the ecological level. Even those that are considered as its most important determinants, such as the entomological situation, living conditions, population density and group immunity, they are not always clearly identified from a statistical point of view. In other words, the results of the investigations point to controversies and uncertainties. Thus, it is necessary to seek the improvement of vector control technologies, which must be implemented universally in urban centers, as well as the improvements in living conditions, especially with regard to basic sanitation infrastructure.”

“Although our study have found a weak relationship between the risk of being infected by CHIKV and population density, it is necessary to remember that, in general, the diseases transmitted by *Ae. aegypti* are modulated by population density.^28,29^ We observed that the SA of highest CHIKV seroprevalence was a space with crowded residences and substandard slum housing, while the SA with lowest seroprevalence was an area with mostly single story housing, with vacant lots between the residences. Specifically, the first one has higher population density than the second. This observation is consistent with knowledge about dengue, whose studies demonstrated a correlation between the prevalence of DENV infection and population density.^10,29,30^ In turn, we also found SA with both relatively high (31.1% - SA 15) and low (2.0% - SA 29) seroprevalence levels with similar population density, 5,631 inhab/km^2^ and 5,865 inhab/km^2^, respectively.”

Please check line 348 for double negative.

Thanks. We corrected it.

The authors declare that their study has “limitations” (line 367), however, they only present one in relation to refusals without further development of the implication(s) of such bias (i.e., selection bias) to the results or their generalizability.

We detailed more information about limitations.

“Our study has limitations due to the refusal to participate (22.1%) that can difficult the generalization of the results. As the refusal was mainly of men, it could have contributed to the encounter of greater seroprevalence among women. Besides that, we also did not investigate other factors that modulate the infections by arboviruses, as the availability of habitats for the vector or other environmental factors that influenced the abundance of *Aedes* in the houses; the seroprevalence of other arboviruses in the same time and space that can lead to competition between arboviruses. However, we understand that our investigation is relevant because it showed the transmission of CHIKV in a naive population, in a scenario of co-circulation with more two arboviruses that infect the same vector, in several intra urban spaces of the city less than three year before the emergence of this agent.”

Reviewer #1:

All figures very poor in resolution and need to be re-draw to increase the clearness and match the required resolution.

We re-drew the figures with better resolution.

Since the title of the manuscript sounds to explore the association of CHIKV seroprevalence with the living condition, it will helpful to present a scatter plot data of each SA versus ILC strata (or even ILC original value, the sum value of all variables). It also good to present the comparison of seroprevalence rate of each ILC strata in boxplot.

Thanks for your suggestion. We elaborated scatter plots about seroprevalence and LCI (sum of all variables) for the four LC strata. Following the other suggestion, we elaborated box plot graphs about seroprevalence rates by LC strata. We add the graphs in results section.

Authors did not consistent in positioning ‘mean’ for each LCI strata. In table 1 it was put at the top of the column while in table 2 was at the bottom of the column for each stratum.

We corrected table 2.

Reviewer #1:

Line 67: Prefer to expand LC to be living condition

We expanded it.

Line 84: Better to mention completely the name of the city in Summary.

We expanded it.

Line 119: better to re-write the number as “0.4% to over 75%”, instead of “over 75% to 0.4%”

We corrected it.

Line 146: write the complete name of IBGE or make a citation to reference #14.

We wrote the complete name of IBGE.

Line 169-170: How this range of population size of each SA was determined?

Thanks for your question. We noticed a mistake in the sentence: “As CT varied greatly in their population size, it was necessary that each SA would have minimum 773 and maximum 2,442 people.” The correct is: “As CT varied greatly in their population size, the SA had minimum 773 and maximum 2,442 people, according to IBGE data.”

Line 170: …each SA had one, two or three CT (Figure 1C). This is not clear in the Figure.

Thanks for warning about it. The Figure 1C did not present this information. We removed figure 1C from Methods section and add in results section, where we present the seroprevalence.

Line 308-312: Was any statistical correlation analysis been performed between ILC strata with seroprevalence rate of the CHIKV? (eg. Spearman correlation test).

Thanks for your suggestion. We performed the Spearman correlation test and did not find any significant statistical correlation. We add this information in the results section.

Line 359-360: Please show the statistical analysis using the study data to support the claim.

Thanks for your suggestion. We removed this claim from discussion and commented that the correlation was weak. Statistical data about this correlation were showed in results section.

“There was negative correlation, no significant (r =-0.18; p= 0.34), between CHIKV seroprevalence and PI, whereas to population density it was positive and significant (r =0.24; p=0.02). Even removing the highest seroprevalence, the population density was still associated (r=0.24 and p=0.03).”

“A finding that stands out in our study is the heterogeneity of the magnitude of CHIKV seroprevalence in intra-urban spaces in the city of FSA, indicating that several factors, beyond living conditions, played a role in the occurrence of this infection, as example the human population density that was correlated with CHIKV seroprevalence, although weakly.”

Line 369-370: Please back up this statement with statistical test as mentioned above (line 308-312 and Line 359-360).

You are right. We included this information in the indicated lines.

Reviewer #2:

Teixeira et al. designed and conducted a population-based, chikungunya virus (CHKV) seroprevalence study in Feira de Santana, Bahia-Brazil. The design of the study and the execution of both field and laboratory procedures in such large sample size are some of the main strengths of the survey, although there are missing details in the description of the methods that should be addressed before publication, particularly related to sampling and incorporation of its complexity in the analysis. Perhaps the main weakness of the study is not related to issues of design or execution but the description of the methodology and the discussion of findings. I strongly encourage the authors to accomplish a more exhaustive review of alternative explanations to the observed high intraurban seroprevalence heterogeneity.

Once again we thank the reviewers for the valuable suggestions, which have been fully accepted throughout the manuscript.

Data Requirements:

Please note that, as a condition of publication, PLOS' data policy requires that you make available all data used to draw the conclusions outlined in your manuscript. Data must be deposited in an appropriate repository, included within the body of the manuscript, or uploaded as supporting information. This includes all numerical values that were used to generate graphs, histograms etc.. For an example see here: <http://www.plosbiology.org/article/info%3Adoi%2F10.1371%2Fjournal.pbio.1001908#s5>.
We attached the the database among other documents for submission.
