# Supplementary material for: Seroprevalence of Chikungunya virus and living conditions in Feira de Santana, Bahia-Brazil
Source: PLoS Negl Trop Dis. 2021 Apr 20;15(4):e0009289. doi: 10.1371/journal.pntd.0009289 (PMC8087031; doi:10.1371/journal.pntd.0009289)
Supplement: Attachment — Submitted filename: resposta aos revisores ok.docx [file pntd.0009289.s005.docx]

**Author's Responses to Reviewers**

**Methods**

Reviewer #1: Method section was improved

Reviewer #2: Authors made changes to the section "Areas Selection" in response to one of my comments, however, there is one difference between the text submitted to the "Reviewer's Responses to Questions"(p. 36; "The Municipal Health Secretariat of Feira de Santana has a wide network...") and the corresponding line 181 in the final manuscript: "The Municipal Health Secretariat of Feira de Santana has an extemsive network..." (there is also a typo). In addition, there are two abbreviations (UBS, line 192; IBGE, line 197) not spelled out in the text.

Thanks for your observations. We corrected the words and spelled the abbreviations in the text: wide network (line 181), BHU (line 192) and IBGE (lines 197-198).

The authors did improve the description of sample size calculation and offered an estimate for the "design effect" of 1.42 (line 224), however, I suggest to clarify whether "rho" in the footnote to table 2 corresponds to an intraclass correlation coeffient (ICC), and if so, to include this estimate in the text.

Thanks for your suggestion. We replaced the “rho” in the footnote of table 2 by “ICC” and included in data analysis in Methods section (lines 249-259):

“We estimated the crude seroprevalence by SA and presented the respective Confidence Intervals of 95%. The global seroprevalence, by LC strata and by sex were estimated and presented the respective Confidence Intervals of 95%, design effect and the intra class correlation (ICC). The Seroprevalence Ratio by LC was estimated as well as the respective Confidence Intervals of 95%. Pearson chi squared (χ^2^) tests were used to test association between CHIKV seroprevalence and the four LC strata assuming p<0.05 and Poisson Regression was used to estimate the Seroprevalence Ratios, assuming as reference the high LC. To test the correlation between CHIKV seroprevalence and ILC, population density and PI, we applied the non-parametric Spearman correlation test.  All analyses were performed using Stata software version 12.0 (Stata Corporation, College Station, USA).”

**Results**

Reviewer #1: Results has been improved but interpretation of the results should stated clearly

Thanks. We improved the interpretation on discussion section (lines 377-392):

“A finding that stands out in our study is the heterogeneity of the magnitude of CHIKV seroprevalence in intra-urban spaces in the city of FSA, indicating that beyond LC, other factors played a role in the occurrence of this infection, as the human population density that was correlated with CHIKV seroprevalence, although weakly. It was notable that there was only a statistically significant difference between high and intermediate LC strata and no difference was observed for the low and very low LC strata, perhaps due to insufficient “n” sampling. However, the average seroprevalence of the high LC stratum was much lower than that of all other strata, indicating that possibly LC play an important role in the dynamics of transmission of this arbovirus in intra-urban spaces. In turn, it cannot be disregarded that the epidemiological cycle of CHIKV involves *Aedes aegypti*, a highly competent vector and, consequently, there is a close relationship between agent transmission and the abundance of winged forms of this mosquito in each intra-urban space. Not by chance, conflicting results on this relationship were also found in a systematic review of the relationship between dengue and poverty^23^ and specifically to chikungunya, this relation was highlighted as controversial in another study.^24^”

Reviewer #2: The authors did provide confidence intervals to selected point estimates of prevalence. Please, clarify why in table 2, according to superscript 3, there was no statistically significant difference between high and other living conditions strata, although in table 3 the 95%IC for the prevalence ratio between intermediate and high LC did exclude the unity: 1.27 - 4.54.? Is this because the intermediate, low and very low LC were pooled together as it might be inferred from lines 306-307?. If pooled, what was the reason to do so? I suggest the authors to restrain from conducting correlation analysis, particularly based on a coefficient correlation, to explore associations between seroprevalence and LC, population density or PI. This could be better approached by using regression analysis, for example, Poisson or negative binomial regression of counts with population as exposure or offset variable.

Thanks for your observations. We rewrite the results to clarify that table 2 and table 3 show different information. The superscript 3 of table 2 was about the association test between CHIKV seroprevalence and LC (Pearson chi squared), while table 3 showed the result of seroprevalence ratio between LC strata, considering high LC as a reference category in Poisson regression.

To clarify this differences, we add the information about Poisson regression on Methods section (lines 253-256):

“Pearson chi squared (χ^2^) tests were used to test association between CHIKV seroprevalence and the four LC strata assuming p<0.05 and Poisson Regression was used to estimate the Seroprevalence Ratios, assuming as reference the high LC.”

We removed the superscript 3 of table 2 and let the result only in the text and replaced the following phrases in results section (lines 312-313):

“The association between the seroprevalence and LC of SA was not significant (Pearson chi squared, p=0.15). The ICC was estimated in 0.20 (CI95% 0.17 – 0.23) (Table 2).”

**Conclusions**

Reviewer #1: From what we see of the fig 4 and 5 and also the correlation test, it was clear that there is no significant correlation of CHIKV seroprevalence and the living condition, it should stated in conclusion or summary explicitly, while suggesting another way or study to measure the risk factors

Thanks. We improved the conclusions based in your suggestions (lines 458-475):

“In summary, despite some limitations of our study, its results indicate that the LC also contribute for the occurrence of CHIKV infections, along with population density. Anyway, it is surprising the no association between the seroprevalence ratios of the low and very low LC and high LC strata, also as well as the failure to find a correlation between vector infestation levels and CHIKV seroprevalence. However, it is already known that the factors that influence the risk of urban arbovirus infections are highly complex at the ecological level. Controversies and uncertainties still remain, highlighting the need for further studies capable to identify the role of each of the determinants that influence the transmission force of the three agents alone or together, especially in the context of a triple epidemic of urban arboviruses, as occurred in FSA. Finally, it is necessary to seek the improvement of vector control technologies, which must be implemented continuously and universally in urban centres affected by these arboviruses, as well as the improvement of LC, particularly with regard to basic sanitation. Initiatives aimed at the development of drugs against chikungunya, reduction of sequelae and deaths are fundamental and, above all, a vaccine to prevent the occurrence of new infections since the vector control measures that have been applied for many decades have not been very effective.”

Reviewer #2: I have no further comments on this regard.

**Editorial and Data Presentation Modifications**

Use this section for editorial suggestions as well as relatively minor modifications of existing data that would enhance clarity. If the only modifications needed are minor and/or editorial, you may wish to recommend “Minor Revision” or “Accept”. 

Reviewer #1: has been suggested in text and example graphs are attached.

Thanks for your suggestion. We considered them and elaborated new graphs that are attached.

Reviewer #2: I have no further comments on this regard.

**Summary and General Comments**

Use  this section to provide overall comments, discuss strengths/weaknesses of the study, novelty, significance, general execution and scholarship. You may also include additional comments for the author, including concerns about dual publication, research ethics, or publication ethics. If requesting major revision, please articulate the new experiments that are needed.

Reviewer #1: This was important public health study to show how living condition may influence risk of get CHIKV infection, however the conclusion taken not clearly show how it influence or not.

Thanks for your observation. We improved the conclusion and the Summary (lines 478-485):

“There is considerable heterogeneity in the occurrence of CHIKV infections in intra-urban spaces of FSA, being higher in more deprived areas, suggesting that the reduction of the incidence of this disease, until safe and effective vaccines are available, strongly depends on the improvement of the living conditions of the population. This improvement should include, mainly, regular water supply, care with the accumulation of trash, greater access to information, households with less crowding and improve the level of education of population. That is, characteristics that can reduce the exposure to risk factors for CHIKV infection.”

Reviewer #2: I have no further comments on this regard.
